# Oral hydrogel nanoemulsion co-delivery system treats inflammatory bowel disease via anti-inflammatory and promoting intestinal mucosa repair

**DOI:** 10.1186/s12951-023-02045-4

**Published:** 2023-08-18

**Authors:** Fenting Lei, Fancai Zeng, Xin Yu, Yiping Deng, Zongquan Zhang, Maochang Xu, Nianhui Ding, Ji Tian, Chunhong Li

**Affiliations:** 1 Analysis and Testing Center, School of Pharmacy, Southwest Medical University, 1-1 Xianglin Road, Luzhou, 646000 Sichuan People’s Republic of China; 2 Laboratory of Biochemistry and Molecular Biology, School of Basic Medical Sciences, Southwest Medical University, Luzhou, 646000 China; 3 Chinese Pharmacy Laboratory, School of Pharmacy, Southwest Medical University, Luzhou, Sichuan China; 4 Department of Pharmaceutical Sciences, School of Pharmacy, Southwest Medical University, 1-1 Xianglin Road, Luzhou, 646000 Sichuan People’s Republic of China; 5 School of Pharmacy, Southwest Medical University, Luzhou, 646000 China

**Keywords:** Curcumin, Emodin, Nanoemulsion, Hydrogel, Inflammatory bowel disease

## Abstract

**Background:**

Due to oral nano-delivery systems for the treatment of inflammatory bowel disease (IBD) are often failed to accumulated to the colonic site and could not achieve controlled drug release, it’s urgent to develop a microenvironment responsive drug delivery to improve therapy efficacy. Inflammation at the IBD site is mainly mediated by macrophages, which are the key effector cells. Excessive inflammation leads to oxidative stress and intestinal mucosal damage. The use of curcumin (CUR) and emodin (EMO) together for the treatment of IBD is promising due to their respective anti-inflammatory and intestinal mucosal repair effects. In view of the pH gradient environment of gastrointestinal tract, here we prepared pH-responsive sodium alginate (SA) hydrogel-coated nanoemulsions to co-deliver CUR and EMO (CUR/EMO NE@SA) to achieve controlled drug release and specifically target macrophages of the colon.

**Results:**

In this study, a pH-responsive CUR/EMO NE@SA was successfully developed, in which the CUR/EMO NE was loaded by chitosan and further crosslinked with sodium alginate. CUR/EMO NE@SA had a pH-responsive property and could achieve controlled drug release in the colon. The preparation could significantly alleviate and improve the colon inflammatory microenvironment by decreasing TNF-α and IL-6 expression, increasing IL-10 expression, scavenging reactive oxygen species in macrophages, and by ameliorating the restoration of intestinal mucosal tight junction protein expression. Furthermore, we revealed the molecular mechanism of the preparation for IBD treatment, which might due to the CUR and EMO synergic inhibition of NF-κB to improve the pro-inflammatory microenvironment. Our study provides a new IBD therapy strategy via synergically inhibiting inflammatory, repairing mucosal and clearing ROS by pH-sensitive hydrogel-encapsulated nanoemulsion drug delivery system, which might be developed for other chronic inflammatory disease treatment.

**Conclusions:**

It’s suggested that pH-sensitive hydrogel-coated nanoemulsion-based codelivery systems are a promising combinatorial platform in IBD.

**Graphical Abstract:**

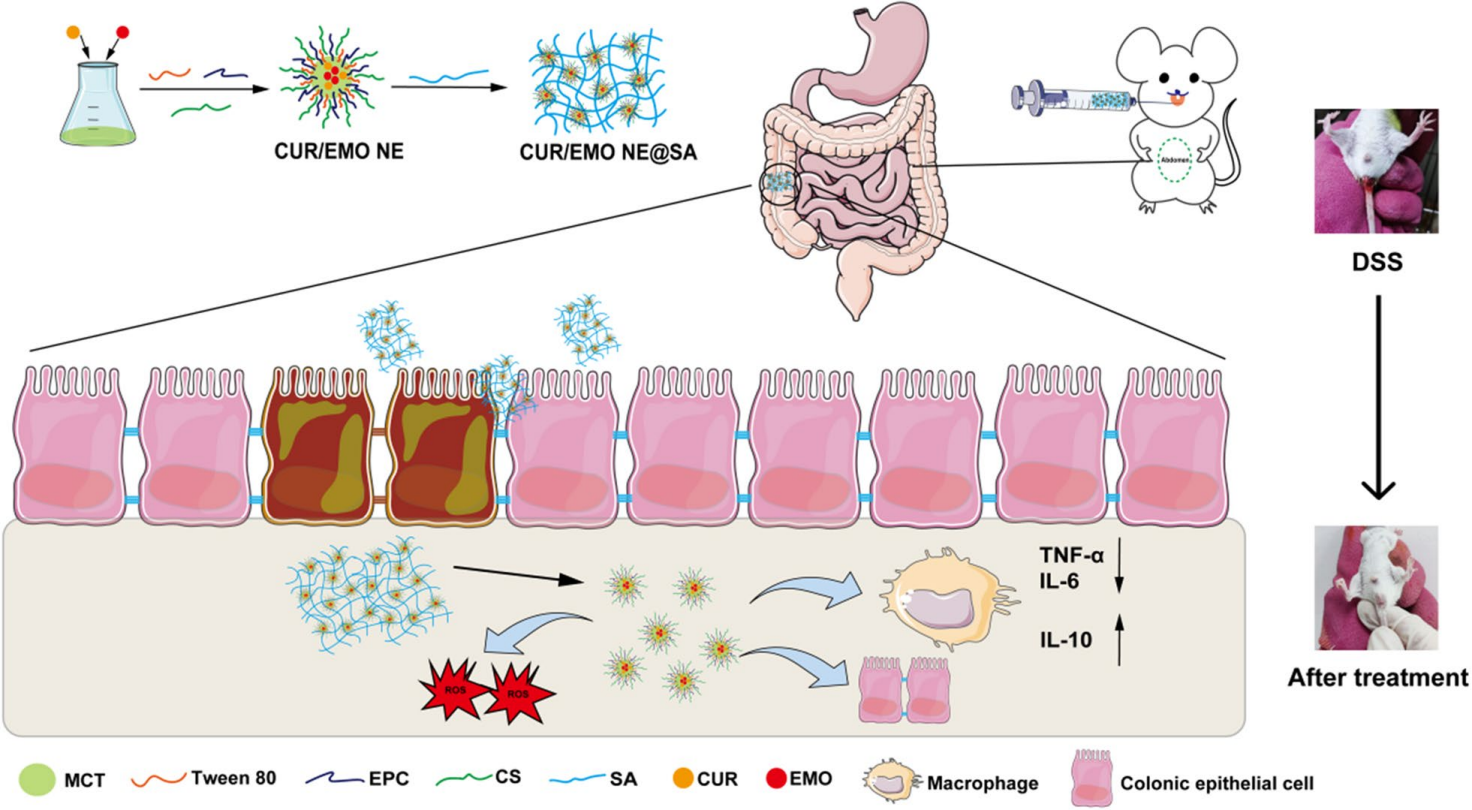

**Supplementary Information:**

The online version contains supplementary material available at 10.1186/s12951-023-02045-4.

## Introduction

Inflammatory bowel disease (IBD) is a chronic, relapsing disease of the colon characterized by diarrhea, rectal bleeding, and weight loss [1]. However, the exact pathogenesis of IBD is unknown, and it is possible that environmental factors, genetic factors, intestinal flora and the immune system are involved in the pathophysiological process of disease [2]. The intestinal microenvironment consists mainly of intestinal epithelial cells, macrophages, dendritic cells, regulatory T cells, and inflammatory T cells, which act together to maintain immune homeostasis [3]. In particular, macrophages play an important regulatory role in intestinal homeostasis. IBD sites are subjected to stimulated polarization of macrophages to M1 type due to damage to intestinal tissues, which continuously release pro-inflammatory factors [interleukin (IL)-6, IL-1β], tumor necrosis factor alpha (TNF-α), and excessive pro-inflammatory factors further aggravate intestinal mucosal damage, excessive oxidative stress, etc. [4], resulting in nonspecific inflammation, mucosal barrier breakdown, and excessive oxidative stress in disease sites [5]. Therefore, macrophages are key effector cells in mitigating the progression of inflammation in IBD [6].

Conventional treatment of IBD is limited to the use of anti-inflammatory drugs, immunosuppressive agents or monoclonal antibodies to reduce the inflammation. In particular, the anti-allergic and immunosuppressive effects of corticosteroids play an important role in the treatment of inflammation, but long-term use of these drugs may be accompanied by complications such as hypertension, diabetes, low bone mass and osteoporosis. In addition, few drugs have both anti-inflammatory and mucosal repair effects. We propose a dual-drug combination treatment strategy aimed at simultaneously inhibiting inflammation and mucosal repair and reducing oxidative stress to alleviate IBD [7].

Curcumin (CUR) has significant anti-inflammatory properties [8–12], which reduces the levels of pro-inflammatory cytokines TNF-α and IL-6 and elevates the levels of anti-inflammatory cytokine IL-10 [13]. It also scavenges reactive oxygen species (ROS) and alters intestinal permeability, effectively relieving colitis [14–16]. Emodin (EMO) is an effective agent for the treatment of IBD with both anti-inflammatory and mucosal repair effects by significantly up-regulating the level of tight junction protein genes such as zonula occludens 1 (ZO-1) and occludin [17–19]. Therefore, combined use of CUR and EMO may be a promising therapeutic strategy through synergistic anti-inflammatory, mucosal repair and counteracting oxidative stress.

Currently, the main delivery system for IBD is oral. However, free CUR and EMO are insoluble monomers with poor water solubility, and oral administration of free CUR and EMO causes rapid metabolic excretion making it difficult to achieve colon-targeted enrichment, limiting their clinical application. To promote drug accumulation at the site of inflammatory bowel disease, researchers are focusing on nano-delivery strategies for the treatment of IBD [20–22], such as pH-sensitive curcumin liposomes or nanoemulsions [23]. Nanoemulsions can improve the aqueous solubility of lipophilic drugs and protect drugs that are susceptible to hydrolysis and oxidation, thereby improving the bioavailability of drugs [24, 25], so co-delivery CUR and EMO via nanoemulsion is advisable.

However, after oral administration, the nanoemulsion readily decomposes in the stomach and small intestine, with subsequent drug leakage, and targeted drug release in the colon cannot be achieved [26]. The pH range of the stomach is low (1.0–3.0), but it is high in the small intestine and colon (5.5–7.5) [27]. It’s reported that hydrogels are a promising drug system due to its colon-targeting property [28–31]. Sodium alginate (SA) is a natural polysaccharide polymer and chitosan (CS) is a natural cationic polysaccharide [32], which can be physically cross-linked to form a three-dimensional hydrogel network [33, 34] with good biodegradability. The hydrophilic three-dimensional spatial network structure of hydrogels can be used to carry nanoemulsions, which are suitable for colon-targeted drug delivery [35–37]. Currently, studies on hydrogel-coated nanoemulsions are more focused on skin diseases, ophthalmic diseases [38, 39], and less studies on oral treatment of inflammatory bowel disease.

**Scheme 1:**
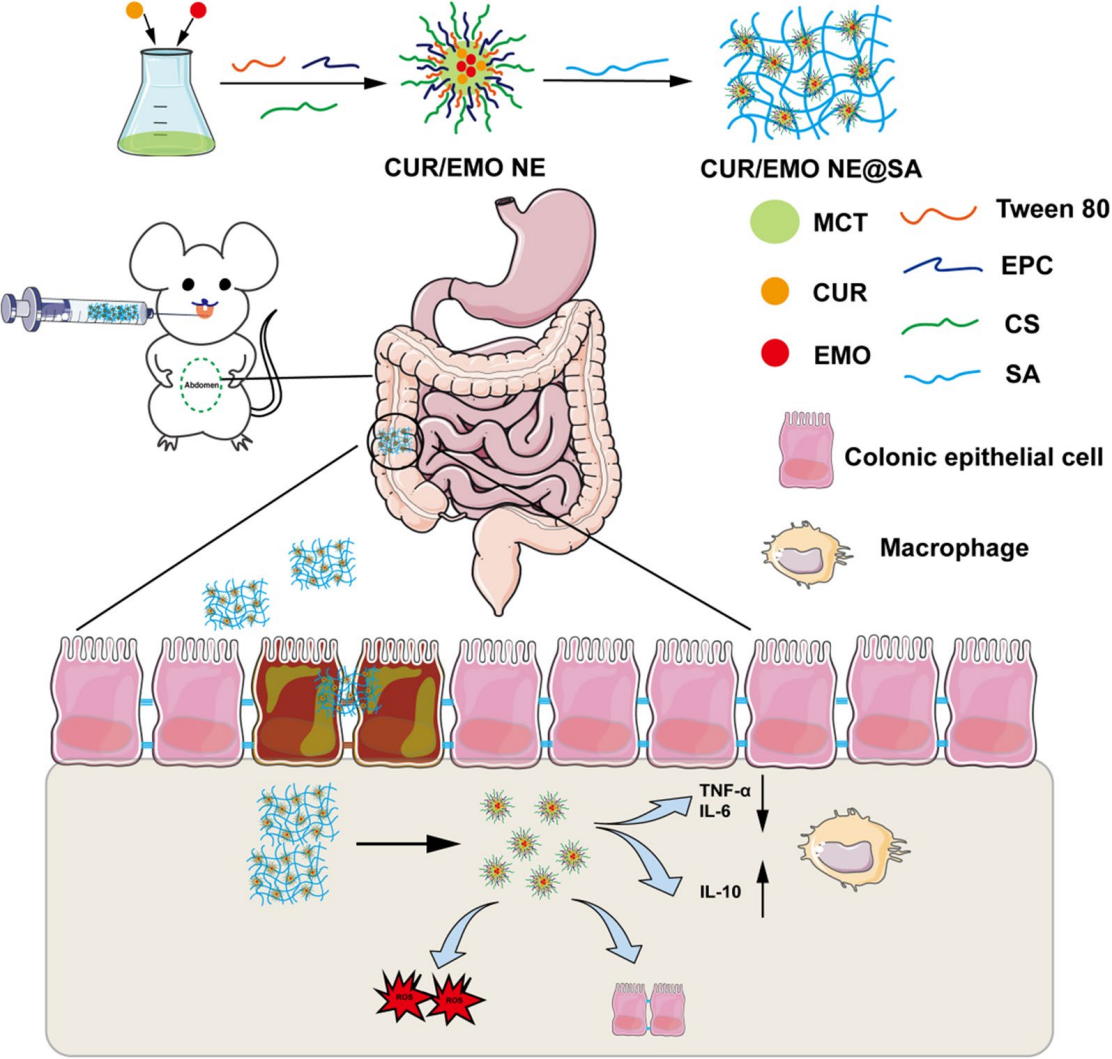
Schematic of preparing CUR/EMO NE by high-energy emulsification and using the physical cross-linking of chitosan and sodium alginate for CUR/EMO NE@SA, as well as demonstrating the advantages of CUR/EMO NE@SA in alleviating IBD

Here, we prepared a pH-sensitive hydrogel-encapsulated nanoemulsion delivery platform (CUR/EMO NE@SA) by first preparing CUR/EMO NE and then coating it using CS and SA cross-linking. In vitro release assays showed that CUR/EMO NE@SA has pH-responsive controlled release properties. In vitro and in vivo pharmacodynamic assays showed CUR/EMO NE@SA mainly target macrophages and achieve anti-inflammatory effects by inhibiting the secretion of pro-inflammatory factors and increasing the secretion of anti-inflammatory factors. In addition, in vivo pharmacodynamic assays showed that CUR/EMO NE@SA had good intestinal mucosal repair efficacy, which could increase expression of ZO-1 and occludin and the epithelial barrier structure was more complete. Our study provides a new strategy for pH-responsive hydrogel-encapsulated nanoemulsion co-delivery system for the treatment of IBD through synergistic anti-inflammation and promotion of intestinal mucosal repair (Scheme 1).

## Materials and methods

### Materials

Curcumin (purity ≥ 98%), Emodin (purity ≥ 98%) and medium chain fatty acids (MCT) was obtained from Shanghai Yuanye Biotechnology Company (Shanghai, China). Egg yolk lecithin, Tween 80, Sodium alginate and Chitosan was obtained from Macklin (Shanghai, China). Dulbecco’s modified eagle’s medium (DMEM), and fetal bovine serum (FBS) were obtained from Gibco (Waltham, USA). Penicillin/streptomycin was obtained from Sola Bioscience. 3-(4, 5-dimethylthiazol-2-yl)-2, 5-diphenyltetrazole bromide (MTT), lipopolysaccharide (LPS) was provided by Solarbio Science & Technology (Beijing, China). Dexosan sulfate sodium salt (DSS, MW 36,000–50,000) was purchased from Yeasen. The enzyme-linked immunosorbent assay (ELISA) kits of TNF-α, IL-6, and IL-10 were purchased from Andygene (Guangzhou, China). All other chemical agents and solvents were analytical grade or above.

The preparation methods of simulated gastric fluid (SGF), simulated small intestine fluid (SIF) and simulated colonic fluid (SCF) were as previously reported. Simply put, dissolve PBS to get buffer. Dilute hydrochloric acid was used to adjust PBS buffer to pH 1.2 to obtain SGF buffer. PBS buffer was adjusted by NaOH to pH 6.8 to obtain SIF buffer. The pH of PBS buffer was adjusted to 7.8 by NaOH to obtain the SCF buffer.

### Cell lines and animals

RAW264.7 cells (Chinese Academy of Sciences, Shanghai, China) were cultured in DMEM supplemented with 10% (v/v) FBS and 1% (v/v) penicillin/streptomycin at 37℃ in an atmosphere containing 5% CO_2_. Colonic carcinoma cells (Caco-2, epithelial properties) (Chinese Academy of Sciences, Shanghai, China) were cultured in DMEM supplemented with 20% (v/v) FBS and 1% (v/v) penicillin/streptomycin at 37℃ in an atmosphere containing 5% CO_2_.

Male Balb/c (20 ± 2 g) were provided by laboratory animal center of southwest medical university (Luzhou, China). All the experimental mice were fed standard diet in normal animal room at temperature of 25 ± 2 °C and relative humidity of 50 ± 10%. In the whole animal experiment, we follow the animal welfare ethical review guidelines in accordance with national standards.

### Preparation of CUR/EMO NE@SA

The nanoemulsion was prepared by high-energy emulsification method. CUR and EMO were added to MCT [40, 41], vortex-mixed and sonicated for 10 min to obtain oil phase containing drugs. The oil phase containing CUR and EMO, egg yolk lecithin, tween 80 magnetic stirring evenly, adding the right amount of chitosan aqueous solution magnetic stirring, then we obtained colostrum [42]. CUR/EMO NE was formed by cell crusher with power of 300 W and crushing time of 10 min (on for 2 s and off for 3 s by ultrasound). Dissolve sodium alginate by stirring and heating with water bath heated magnetic stirrer to obtain homogeneous 2% SA, which were mixed NE equably at 100 rpm to obtain hydrogel nanoemulsion (CUR/EMO NE@SA). Blank NE without any drug and CUR NE, EMO NE for drug were prepared by the same method.

### Anti-inflammatory and mucosal repair of CUR and EMO

In order to make CUR/EMO NE@SA exert better in anti-inflammatory and mucosal repairing, we screened the ratio of CUR and EMO from two ways [43, 44]. Since activated macrophages play an important role in the progression of IBD, we used them as model cells to evaluate the anti-inflammatory effects of CUR and EMO. To further investigate the anti-inflammatory effects of CUR and EMO, we decided to use quantitative Real-Time Polymerase Chain Reaction (qRT-PCR) to detect the expression of pro-inflammatory cytokines TNF-α and IL-6 and anti-inflammatory cytokine IL-10 to determine the ratio and dose of the two drugs. RAW264.7 cells were placed on the 6-well plate (2 × 10^5^ per well). After the cells were attached to the wall, LPS was added for stimulation and activation for 12 h. EMO (10 μg/mL) and CUR (0, 5, 10, 15, 20 μg/mL) or CUR (10 μg/mL) and EMO (0, 5, 10, 15, 20 μg/mL) were added into cell culture pore plates, co-culturing 10 h. Trizol method was used to extract total RNA from cells, and reverse transcription was performed. Non-treatment and LPS groups were used as controls. Moreover, to refine the screening ratios even more, we detected TNF-α expression at different ratios by qRT-PCR at a total drug concentration of 20 μg/mL. To make it clear, we also performed TNF-α experiments with western blot (WB), aiming to combine the results of qRT-PCR for better screening of drugs ratio.

We also screened drug ratio from mucosal repair, which could be indicated by wound healing assay [45]. Caco-2 cells were cultured to 80% in 6-well plates and wounds were scraped with the tip of 200 μL disposable pipette. Then CUR (20 μg/mL), EMO (20 μg/mL) and CUR/EMO (10/10 μg/mL) were co-incubated with the cells for 24 h and 48 h. The wound healing process was evaluated using a microscope at different time points. Particularly, the mucosal recovery ability of the drug was investigated in caco-2 cells by detecting tight junction proteins, so we used qRT-PCR to detect the RNA expression of olaudin-1 [46, 47].

### Inhibition of NF‑κB activation by CUR and EMO

Furthermore, in order to investigate the anti-inflammation signaling pathway mechanism of CUR and EMO. We divided them into control (without LPS), LPS, Cur + LPS, EMO + LPS and Cur/EMO + LPS groups, and studied the protein levels of nuclear factor-κB (NF-κB) p65, phosphorylated NFкB p65 (p-NFкB p65), IκBα, and phosphorylated IκBα (p-IκBα) by western blot [19, 48].

### Characterization of CUR/EMO NE@SA

Encapsulation efficiency (EE) and drug loading efficiency (DLE) were determined by high performance liquid chromatography (HPLC). The CUR/EMO NE and CUR/EMO NE@SA were placed in an ultrafiltration centrifuge tube at 4000 rpm for 10 min. After centrifugation, the ultrafiltration device was removed from the centrifuge and filtrate was collected. The 500 μL preparation was dissolved in methanol and demulsified by ultrasound for 10 min. The above solution was diluted with methanol, and the free drug and total drug contents were determined by HPLC. The difference values could be used to calculate the EE and DLE.

Particle size, polydispersity index (PDI) and zeta potential of the CUR/EMO NE and CUR/EMO NE@SA were measured at 25℃ by Malvern laser particle size meter. Nanoemulsion and hydrogel were also analyzed by transmission electron microscope (TEM). The sample was diluted with ultra-pure water and stained with phosphotungstic acid for about 30 s. The CUR/EMO NE@SA was lyophilized for 24 h, and scanning electron microscopy (SEM) photographs were taken to observe the 3-dimensional structure.

With regards to the stability, CUR/EMO NE was stored in refrigerator at 4 °C for 1 week and evaluated by regular daily checks of particle size and PDI with Malvern laser particle size meter.

### Rheological properties testing

The rheological properties of the samples were tested using a rotational rheometer (Model: MCR92, from Anton Paar, Austria) [15, 49–51]. An appropriate amount of sample was taken and placed on the sample stage, the model of the test rotor was 50 mm for parallel plates, the gap was set at 1 mm, and the test temperature was 25 °C. Set the strain scanning, scanning range 0.01–1000%, logarithmic point, frequency set to 1 Hz. Set up angular frequency scanning, scanning range 0.1–100 Hz, logarithmic point taking, strain setting 1%. Set time modulus profile, linear scan at 0–10 min, gap 0.6 mm, strain 1%. Set the shear mode with a shear rate of 0.1–100 s^−1^ for the rheological behavior and logarithmic point taking.

### Stability of CUR/EMO NE@SA in gastrointestinal simulations

To examine the pH responsiveness, the CUR/EMO NE and the CUR/EMO NE@SA were measured for cumulative release in simulated gastric fluid (pH 1.2), simulated intestinal fluid (pH 6.8), and simulated colonic fluid (pH 7.8). The samples were loaded into dialysis bags (4000 MW) and then placed in medium liquid simulation at 37℃ and shaken 100 rpm from 0–2 h in SGF, 2–6 h in SIF, and 6–24 h in SCF. Measured at predetermined intervals, 1 mL of each simulated solution was removed and same volume was added. 1 mL of liquid was added into 1 mL of methanol, ultrasonic broken, vortex mixed, and 0.22 μL filtered, and the contents of CUR and EMO were determined by HPLC. Repeat this step 3 times for each sample. The cumulative release of CUR and EMO was calculated according to the formula:

$$Drug\;release\;amount{ }\left( \% \right) = \frac{Drug\;amount\;in\;release\;medium}{{Drug\;load\;of\;nanoparticles}} \times 100$$

In addition, to investigate the structural changes of CUR/EMO NE@SA under different pH conditions to simulate the gastrointestinal microenvironment. We stirred CUR/EMO NE@SA with dialysis bag under different pH (pH 1.2, 6.8, 7.8) for 6 h and then lyophilized it for 24 h respectively, and observed its structure by SEM.

### Cytotoxicity assay

The in vitro biocompatibility of CUR, EMO, CUR/EMO, Blank NE, CUR/EMO NE, CUR/EMO NE@SA were analyzed by MTT. RAW264.7 cells at logarithmic growth stage were inoculated in 96-well plates (5 × 10^3^ per well). After 24 h, preparation of 1, 5, 10, 15, 20 μg/mL concentrations (200 μL of per well) were added and incubated for 24 h, following by adding 20 μL of MTT (5 mg/mL) and 180 μL of complete medium to each well and incubating in cell incubator for 4 h. Finally, formazan was dissolved with 150 μL of DMSO. The absorbance at 490 nm, 37 °C was measured by multifunctional enzyme labeler.

The in vitro cytotoxicity of CUR, EMO, CUR/EMO, Blank NE, and CUR/EMO NE were analyzed by Calcein-AM/PI staining. Cells were inoculated into 12-well plates (5 × 10^4^ per well) and placed overnight. Then, preparations with concentrations of 1, 5, 10, 15, 20 μg/mL were added and incubated for 24 h. Staining solution was obtained by adding 5 μL of Calcein AM solution (2 mM) and 15 μL of PI solution (1.5 mM) to 5 mL of buffer solution, which was added to each well and incubated at 37 °C for 30 min, then taking fluorescence microscope pictures. All cytotoxicity assays were performed three times.

### In vitro cellular uptake

Nanoemulsion uptake by RAW264.7 cells was quantitatively detected by confocal laser scanning microscopy (CLSM) and flow cytometry. The preparation of encapsulated fluorescent probe coumarin 6 (C_6_) instead of CUR and EMO was prepared. RAW264.7 cells were inoculated into 24-well plates pre-placed with crawlers (4 × 10^4^ cells per well). After waiting for the cells to attach to the wall, they were divided into control (without LPS) and LPS groups (5 μg/mL) and incubated overnight. The C_6_ NE (1 μg/mL) was diluted with DMEM medium and incubated for 2 h. After 2 h, the preparation was sucked out and washed three times with PBS. The cells were added with 4% paraformaldehyde solution of 500 μL and incubated at room temperature and sheltered from light for 15 min. The paraformaldehyde was sucked and washed with PBS. After washing with PBS, each well was added with 1% FBS (diluted with PBS) and closed at room temperature for 30 min. The cells grow on cover glass were taken out and placed on the slide, then anti-fluorescence quench agent containing DAPI was added, followed by observing under CLSM (430 nm excited) as soon as possible.

RAW264.7 cells were inoculated into 12-well plates (8 × 10^4^ cells per well) with slime placed in advance. After the cells adhered to the wall, they were divided into control (without LPS) and LPS groups (5 μg/mL) and incubated overnight. C_6_ NE was diluted with DMEM medium and co-cultured for 2 h. Then the medium was sucked off, washing cells three times with PBS, and then 200 μL PBS was added to scrape off the suspension cells, and quantitative detection was performed by flow cytometry.

### Effects of CUR/EMO NE on inflammatory factors in vitro

RAW264.7 cells in logarithmic growth stage were digested into single-cell suspension and inoculated into 6-well plates (5 × 10^5^ cells per well), culturing for 24 h. Then LPS was added and cultured for 24 h to activate cells. The medium was replaced with 2 mL various formulations solution including the CUR (20 μg/mL), EMO (20 μg/mL), and CUR/EMO (10/10 μg/mL), Blank NE, and CUR/EMO NE (10/10 μg/mL). After incubation for 10 h, the expressions of pro-inflammatory factors (TNF-α, IL-6) and anti-inflammatory factors (IL-10) were detected by qRT-PCR.

### Intracellular ROS scavenging of CUR/EMO NE in vitro

DPPH (1,1-diphenyl-2-picrylhydrazyl) radical clear assay was used to evaluate antioxidant activity in vitro. A 96-well plate was added with 200 μL DPPH solution (0.04 mM) ethanol, followed by CUR, EMO, CUR/EMO, Blank NE, CUR/EMO NE, CUR/EMO NE@SA dispersed in 20 μL ultra-pure water. Then the solution was incubated in the dark at room temperature for 30 min, and the absorbance of the solution was measured at 517 nm using enzyme label. The DPPH inhibition rate was calculated according to the following formula:

$${\text{DPPH inhibition }}\left( \% \right)\, = \,\left( {{\text{Ac}} - {\text{Ae}}} \right)/{\text{Ac}}\, \times \,{1}00\% .$$

Ac is the absorbance of the control (no sample, only DPPH), and Ae is the absorbance of the sample solution. Each experiment was repeated three times.

RAW264.7 cells were inoculated into 96-well plates (1 × 10^4^ cells per well) and incubated for 4 h, aiming to study the intracellular ROS scavenging of CUR/EMO NE [52]. The medium in each well was replaced with a fresh 200 μL medium containing 100 ng/mL LPS to stimulate ROS production, except that control group was replaced with 200 μL fresh medium without LPS. Subsequently, one group of LPS-treated cells received no treatment and the other groups were treated with the CUR (20 μg/mL), EMO (20 μg/mL), and CUR/EMO (10/10 μg/mL), Blank NE, and CUR/EMO NE (10/10 μg/mL), respectively. After 24 h, the medium was discarded and washed with PBS for three times, and then ROS probe DCFDA (10 μM) was added. After incubation in the dark for 30 min, fluorescence detection (excitation wavelength of 488 nm and emission wavelength of 525 nm) was carried out with enzyme labeler to quantify the intracellular ROS levels.

Similarly, RAW264.7 cells were inoculated 12-well plates (2 × 10^5^ cells per well) and treated with the same procedure described above. After treatment, probe DCFDA was added to detect intracellular ROS, which was an probe that could only be oxidized by ROS to display fluorescence emission. Intracellular ROS localization and levels were monitored via Multichannel fluorescence microscopy (MFM) using FITC channels (488 nm excitation).

### In vivo biodistribution evaluation

DSS (36,000–50,000, molecular weight) was first mixed with 2% DSS drinking water and fed to mice for 5 days, and the model of IBD was established [53]. A single cycle can induce acute IBD. After oral administration of DID NE@SA, the adhesion effect and tissue distribution (heart, liver, spleen, lungs, kidneys, colon) were studied by real-time in vivo fluorescence imaging and in vitro organ imaging [51].

### Evaluation of therapeutic effect on colitis mice

Mice were given 2% DSS for 5 days to induce IBD. The colitis animals were randomly divided into the DSS group and five treatment groups, such as CUR, EMO, CUR/EMO, CUR/EMO NE, CUR/EMO NE@SA, while the healthy group served as the control group. On the third day of modeling, intragastric administration was performed at the doses of 20 mg/kg in CUR, 20 mg/kg in EMO, and 10/10 mg/kg in CUR/EMO, CUR/EMO NE, and CUR/EMO NE@SA.

From the beginning of modeling to the end of treatment, the weight change, disease activity index (DAI) was investigated every day. After administration, the colonic representative pictures and histological analysis of colon were performed. The severity of colitis after drug treatment was evaluated histologically by HE staining of colon tissue, and the infiltration of inflammatory cells was observed.

### In vivo anti-inflammatory and mucosal repair

The contents of pro-inflammatory factors (TNF-α, IL-6) and anti-inflammatory factors (IL-10) were detected by ELISA in mice plasma and colon tissues.

Impairment of epithelial barrier function is typical of the pathophysiology of IBD. In order to detect the influence of each group on IBD epithelial barrier function, we detected the expression of ZO-1 and occludin, two important components of epithelial cytoskeleton, by immunohistochemistry (IHC) [45].

### Preliminary safety evaluation

At the end of the pharmacodynamic experiments, bloods were collected from the eyeball, and the plasma were used for the detection of AST and ALT. The organs were washed with saline, dried with filter paper and weighed for tissue coefficient calculation [54, 55]. The liver, spleen and kidney of mice were sealed with 4% paraformaldehyde for HE staining to evaluate the safety of the preparation in vivo.

### Statistical analysis

Data were statistically analyzed using GraphPad Prism 9 and are expressed as the mean ± standard deviation. Statistical tests were performed among the groups using one-way ANOVA followed by Scheffe’s post hoc multiple-comparison or Student’s t-test was used for two groups. Statistical significance was set at p < 0.05.

## Results

### Anti-inflammatory and mucosal repair of CUR and EMO

We examined the expression of pro-inflammatory cytokines and anti-inflammatory cytokine by qRT-PCR at different ratios and dosages of CUR/EMO acting on activated macrophages. By the results, when CUR was 10 μg/mL, it roughly exhibited a trend of decreasing the expression of TNF-α, IL-6 and increasing the expression of IL-10 as EMO increased from 5 to 20 μg/mL (Additional file 1: Fig. S1A–C). Similarly, when EMO was 10 μg/mL, as CUR was increased from 10 to 20 μg/mL, it broadly exhibited a trend to decrease the expression of TNF-α, IL-6 and increase the IL-10 (Additional file 1: Fig. S1D–F). Therefore, the combination of CUR/EMO could achieve better therapeutic effect, which showed a good dose-dependent anti-inflammatory effect. By ratio screening, we found that the CUR/EMO in proportion of 10/10 μg/mL had best TNF-α lowering effect (Additional file 1: Fig. S2). More importantly, through the western blot experiments, we further verified that in this proportion (10/10 μg/mL) the effect of reducing TNF-α was the most obvious (Additional file 1: Fig. S3).

To further determine the mucosal repair effects of the drugs, we performed in vitro wound healing assay with caco-2 cells. It demonstrated that increased proliferation and migration of caco-2 cells after CUR/EMO treatment compared to free CUR or EMO (Additional file 1: Fig. S4). The combination of CUR and EMO might accelerate mucosal repair. CUR/EMO at 10/10 μg/mL showed the highest expression of olaudin-1 (Additional file 1: Fig. S5A, B). When the concentration ratio of CUR and EMO was 1:1, the expression of olaudin-1 increased with increasing concentration (Additional file 1: Fig. S5C).

In MTT experiments, CUR/EMO NE@SA exhibited good biocompatibility when CUR/EMO was 10/10 μg/mL, which was chosen for subsequent experiments (Additional file 1: Fig. S10). In summary, we screened the CUR/EMO of 1:1 (wt:wt) from biocompatibility, inflammatory factor expression, and mucosal repair.

### Inhibition of NF‑κB activation by CUR and EMO

NF-κB regulates the expression of a large number of cytokines, and its activation plays a role in IBD. NF-κB P65 normally binds to its inhibitor, IκBα, but once stimulated by inflammatory factors (e.g., LPS), IκBα is degraded, eliminating inhibition of NF-κB P65. Then NF-κB P65 is phosphorylated and initiates the expression of inflammatory cytokines, which activate the NF-κB pathway. To determine whether CUR and EMO mediate the activation of NF-κB, we further examined the protein levels of NF-κB p65, p-NF-κB p65, IκBα, and p-IκBα by western blot. The results were shown that the LPS + CUR, LPS + EMO and LPS + CUR/EMO groups significantly inhibited p-NF-κB p65 (Additional file 1: Fig. S6). In addition, the inhibitory effect of CUR/EMO on the NF-κB pathway was significantly better than that of Cur or EMO. CUR and EMO could inhibit the level of p-NF-κB p65/p-IκBα in LPS-induced macrophages and inhibit the activation of NF-κB in LPS-activated macrophages to regulate the inflammatory response.

### Preparation and characterization of CUR/EMO NE@SA

**Fig. 1:**
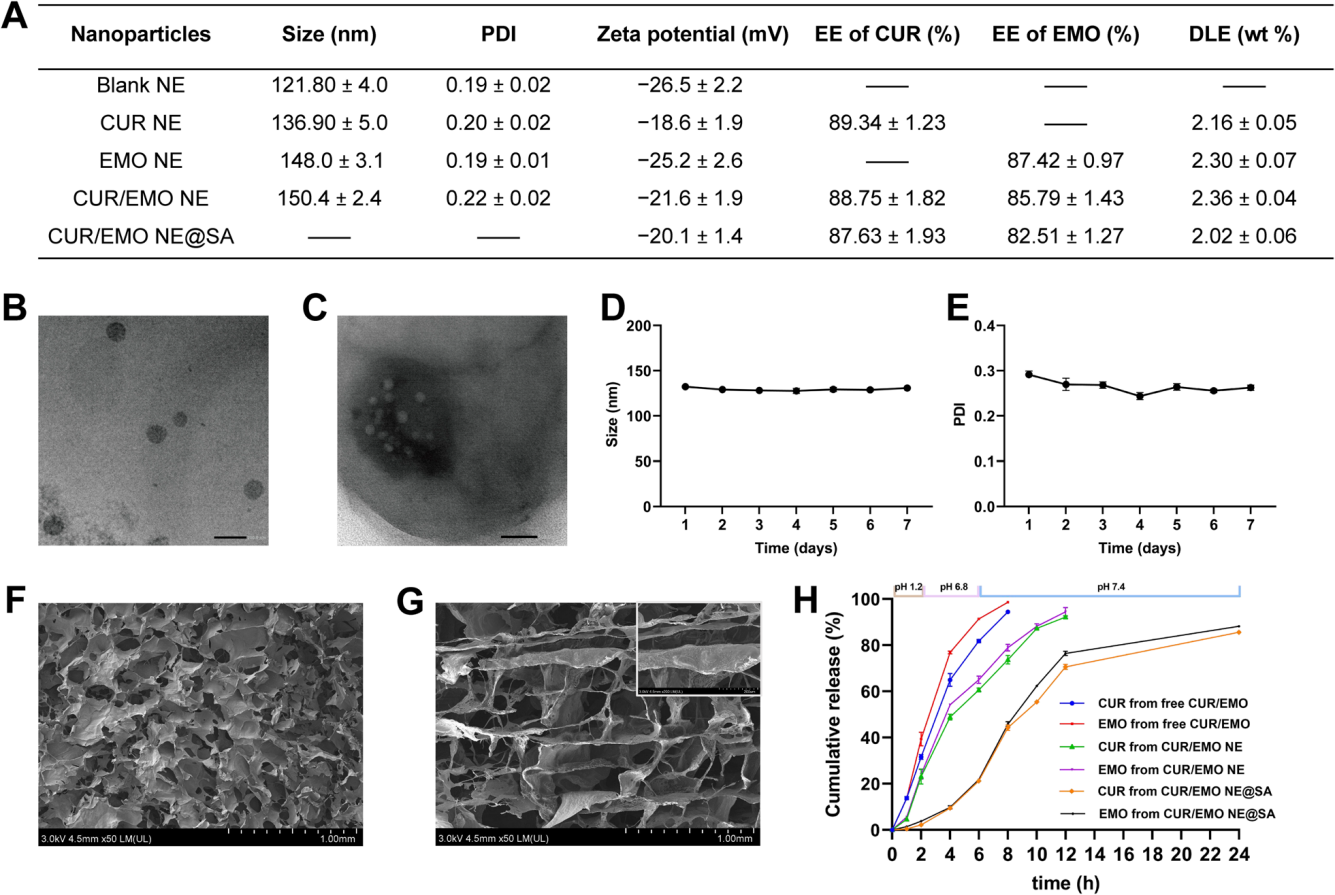
Characteristic of nanoparticles. **A** Size, PDI, zeta potential, EE and DLE of nanoparticles. Data are shown as mean ± SD (n = 3). The transmission electron microscope of **B**, the CUR/EMO NE and **C** the CUR/EMO NE@SA. The scale bar of **B** was 200 nm, and the scale bar of **C** was 500 nm. Stability evaluation of CUR/EMO NE with detection of particle **D** size and **E** PDI at the same time for 7 days (n = 3). Scanning electron microscope of **F** CS/SA hydrogel and **G** CUR/EMO NE@SA. **H** Cumulative release of CUR or EMO from free CUR/EMO, CUR/EMO NE and CUR/EMO NE@SA. Data are shown as mean ± SD (n = 3)

CUR and EMO were dissolved in MCT as the oil phase, egg yolk lecithin as the emulsifier and Tween 80 as the co-emulsifier, and chitosan aqueous solution as the aqueous phase to prepare the nanoemulsion, and then sodium alginate was added to physically cross-link with chitosan to form CUR/EMO NE@SA [56]. The mean hydrodynamic diameter of CUR/EMO NE was 130.4 ± 2.4 nm. The CUR NE was 136.90 ± 5.0 nm, EMO NE was 148.0 ± 3.1 nm. The PDI of all nanoparticles was less than 0.3, indicating uniform size. The mean zeta potential was − 21.6 ± 1.9 mV for the CUR/EMO NE, − 18.6 ± 1.9 mV for the CUR NE, − 25.2 ± 2.6 mV for the EMO NE, and − 26.5 ± 2.2 mV for the blank NE. The drug encapsulation efficiency of the CUR/EMO NE was 88.75 ± 1.82% (w/w) for the CUR, 85.79 ± 1.43% (w/w) for the EMO, and the total drug loading efficiency was 2.36 ± 0.04% (w/w) (Fig. 1A).

TEM images revealed that the CUR/EMO NE was monodisperse and uniformly spherical with dimensions of 100–200 nm (Fig. 1B), and the CUR/EMO NE@SA was successfully wrapped the CUR EMO NE (Fig. 1C). From the SEM images, it could be seen that the hydrogel successfully encapsulated the nanoemulsion, which the void was about 200 μm (Fig. 1F, G). From the stability experiments, the CUR/EMO NE maintained stable particle size and good uniformity of dispersion within 1 week (Fig. 1D, E). In addition, we also took CUR/EMO NE pictures for the first and seventh day, suggesting its favorable stability (Additional file 1: Fig. S7).

### Rheological characterization

Rheological characterization is the study of hydrogel viscoelasticity. In order to evaluate the viscoelasticity of hydrogels, we first performed dynamic strain scans to set up suitable conditions for dynamic frequency scans. In lower strain range (0.0001%–0.01%), the values of the energy storage modulus (G') and the loss modulus (G″) were constant, indicating that it was an undisturbed gel state, and its intersection represented the critical point between gel and fluid, indicating that the sample was a water gel (Additional file 1: Fig. S8A). However, at higher shear strains (> 0.01%), the G' value decreased and at a shear strain of 0.06%, the G' and G″ values cross each other, indicating a transition from gel to sol. It noticed that an increase in stress (> 1 Pa) results in structural instability of the sample (Additional file 1: Fig. S8B). After setting the strain amplitude to 1.0%, a dynamic angular frequency scan was performed. From 0.1 to 100 Hz, the value of G' was several times that of G″, which indicated that the hydrogel was resistant to external forces (Additional file 1: Fig. S8C), but the structure of the hydrogel was altered at high frequencies. Keeping the strain at 1%, the dynamic time scans were performed, and the linear scans were performed in 0–10 min, and the G' were all greater than the G″, and the surface hydrogel had good stability (Additional file 1: Fig. S8D). Finally, the shear dilution process of the hydrogel was investigated by performing a shear mode scan with shear rate of 0.1–100 s^−1^, as shown in Additional file 1: Fig. S8E, F. The viscosity decreased with the increase of the shear rate, and hydrogel thinning effect could be observed, which indicated that when a certain amount of shear stress or pressure was applied to the hydrogel, and the hydrogel was able to flow smoothly through the gavage needle. Combined with the rheological characterization results, we had successfully prepared CUR/EMO NE@SA with certain stability and good gavage.

### Stability of CUR/EMO NE@SA in gastrointestinal simulations

The CUR/EMO NE released the CUR and EMO at slower rate than the free drug solution, with almost 95% of the free CUR/EMO released from the corresponding free drug solution at about 12 h. In the CUR/EMO NE@SA, the cumulative drug release reached more than 90% at 24 h. In addition, as seen from the CUR/EMO NE@SA, there was little drug release in the SGF at pH 1.2 for 2 h. In the SIF with pH of 6.8 for 4 h, the cumulative drug release was about 20%. In the SCF with a pH of 7.8, the cumulative amount of the drug released after 6 h could be released to about 70% (Fig. 1H).

In addition, combined with the SEM of the CUR/EMO NE@SA at different pH, the hydrogels in SGF (pH 1.2) collapsed to form a dense structure, as shown in Additional file 1: Fig. S9A-C. In contrast, the hydrogel in SIF (pH 6.8) underwent swelling, leading to an increase in pore size. The hydrogel in SCF (pH 7.8) further swelled. These results suggest that the CUR/EMO NE@SA might better deliver drugs to the colon.

### Cytotoxicity assay

In order to prove the cytotoxicity on macrophages, we used the MTT method to detect the effects of CUR, EMO, CUR/EMO, Blank NE, CUR/EMO NE, and CUR/EMO NE@SA at different concentrations. From the MTT results, the cell viability of free CUR/EMO on RAW264.7 was lower (about 20%) at 1 μg/mL, indicating its greater cytotoxicity (Additional file 1: Fig. S10). However, the biocompatibility of the drug could be further improved by preparing the formulation, and at 10 μg/mL, CUR/EMO NE showed good biocompatibility (> 80%), and CUR/EMO NE@SA also possessed good biocompatibility (Additional file 1: Fig. S11). In addition, the results of Calcein-AM/PI staining also were in accordance with MTT. Compared to free CUR/EMO, CUR/EMO NE showed better biocompatibility.

### In vitro cellular uptake

**Fig. 2:**
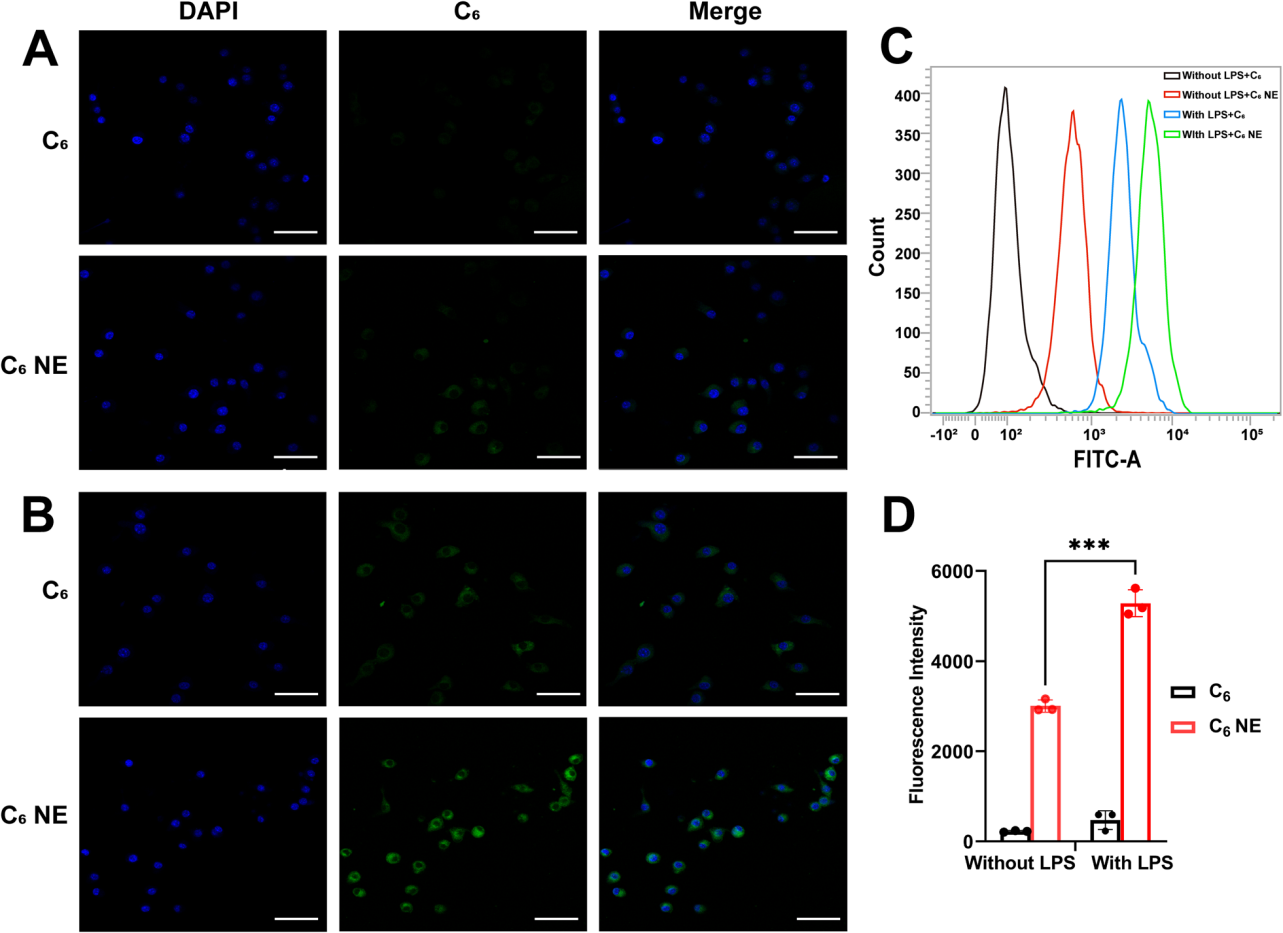
Uptake of C_6_ NE in RAW264.7 cells. After incubating RAW264.7 cells with C_6_ NE for 2 h, the fluorescence intensity was detected by **A**, **B** CLSM images and **C** flow cytometry. Blue: cell nucleus; green: C_6_. **A**: without LPS; **B**: with LPS. The Scale bar were 50 μm. **D** Semi-quantification of the fluorescence intensity. Data are shown as mean ± SD (n = 3). ***p < 0.001

To evaluate cellular uptake of nanoparticles by RAW264.7 cells, encapsulated fluorescent probe C_6_ was prepared instead of CUR/EMO. From the CLSM results, LPS-activated cells (Fig. 2B) internalized more C_6_ NE than inactivated cells (Fig. 2A). And C_6_ NE showed the highest uptake regardless of cell activation, indicating a high affinity for target cells. Flow cytometry also confirmed a higher uptake rate of C_6_ NE than free C_6_ (Fig. 2C, D), consistent with confocal results.

### Effects of CUR/EMO NE on inflammatory factors

**Fig. 3:**
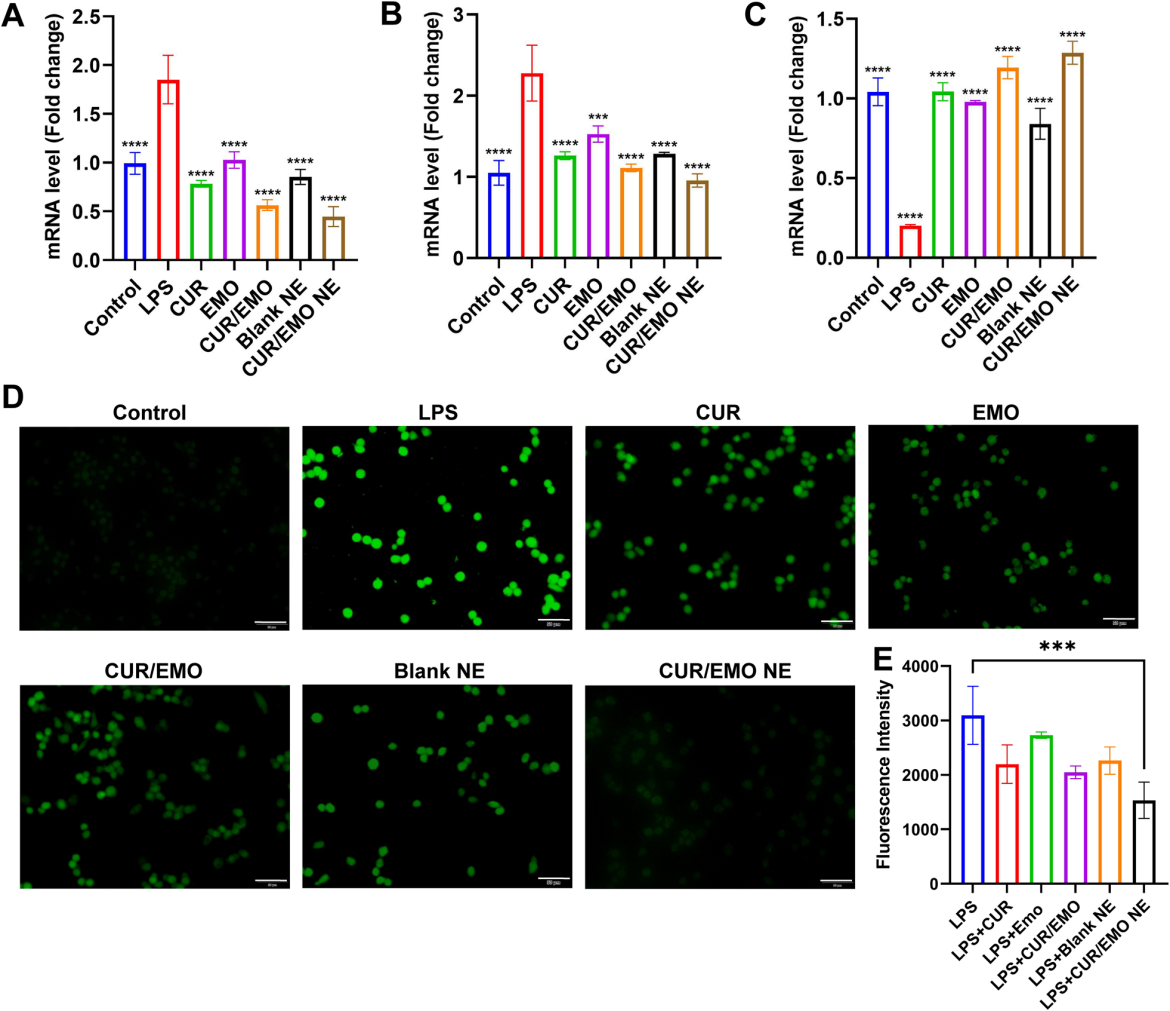
In vitro inflammatory factors and ROS of C_6_ NE in RAW264.7 cells. The expression of TNF-α (**A**), IL-6 (**B**), IL-10 (**C**) was determined by qRT-PCR after treatment of RAW264.7 cells with different preparations stimulated by lipopolysaccharide. Data are shown as mean ± SD (n = 3). *p < 0.05, **p < 0.01, ***p < 0.001, ****p < 0.0001 vs LPS group. **D** CLSM images of RAW264.7 cells with different treatments. Green: ROS. Scale bar were 50 μm. **E** Fluorescence intensity of DCFDA in RAW264.7 cells with different treatments. ***p < 0.001

In order to observe the anti-inflammatory effect of CUR/EMO NE on activated macrophages, the levels of inflammatory factors were determined after different treatments. QRT-PCR method was used to detect the transgress CUR (20 μg/mL), EMO (20 μg/mL), CUR/EMO (10/10 μg/mL), Blank NE, and CUR/EMO NE (10/10 μg/mL) on the expression of pro-inflammatory factors and anti-inflammatory factorsin RAW264.7 cells. The results showed that the levels of TNF-α and IL-6 were lower in CUR/EMO NE group than in other groups, and the levels of IL-10 were higher, suggesting CUR/EMO NE could inhibit the production of pro-inflammatory cytokines and promote the release of anti-inflammatory (Fig. 3A–C).

To explore in more detail how the drugs CUR and EMO combat IBD in activated macrophages, we analyzed the effects of CUR and EMO on levels of inflammatory cytokines. The CUR resulted in significantly lower levels of TNF-α and IL-6 than EMO, and the CUR/EMO resulted in significantly higher levels of IL-10 than the CUR or EMO. These results indicated that both CUR and EMO could decrease TNF-α and IL-6 levels and increase IL-10 levels, suggesting their synergic anti-inflammatory effects.

### Intracellular ROS scavenging of CUR/EMO NE

Previous studies have reported that the CUR has potential anti-inflammatory activity due to the antioxidant activity of its two phenol hydroxyl groups and the beta-dione portion of the enol. To further investigate whether EMO, CUR/EMO, Blank NE, CUR/EMO NE, and CUR/EMO NE@SA have antioxidant activity, we used DPPH radical scavenging assay to evaluate their anti-oxidation ability. CUR, CUR/EMO, CUR/EMO NE, and CUR/EMO NE@SA showed increased scavenging activity on DPPH free radicals with increasing concentration, while EMO and Blank NE showed no scavenging activity on DPPH free radicals, indicating that the co-loading of EMO did not affect the good antioxidant activity of CUR, and that CUR/EMO NE and CUR/EMO NE@SA preparations did not affect their antioxidant activity (Additional file 1: Fig. S12).

Whether the CUR/EMO NE@SA retains its antioxidant activity in complex intracellular environments remains unclear. Therefore, we further performed intracellular ROS clearance experiments in activated RAW264.7 cells to evaluate the intracellular antioxidant activity of CUR/EMO NE [57]. After being treated by the CUR, EMO, CUR/EMO, Blank NE, and CUR/EMO NE, intracellular ROS levels were detected by DCFDA. Each group showed different levels of reduced green fluorescence compared with the LPS group (Fig. 3D), indicating that the preparation had a ROS clearance effect. This was also confirmed by the quantitative analysis in Fig. 3E. EMO showed different results in DPPH and ROS clearance experiment, and this difference could be explained by the different roles of EMO in other detection. However, in intracellular ROS clearance assay, macrophages’ inflammatory response to LPS stimulation was induced. As an anti-inflammatory drug, EMO may inhibit the production of ROS by relieving inflammation, so the CUR/EMO showed better activity in clearing intracellular ROS due to the synergistic effect of the CUR’s antioxidant activity and anti-inflammatory activity.

### In vivo biodistribution evaluation

**Fig. 4:**
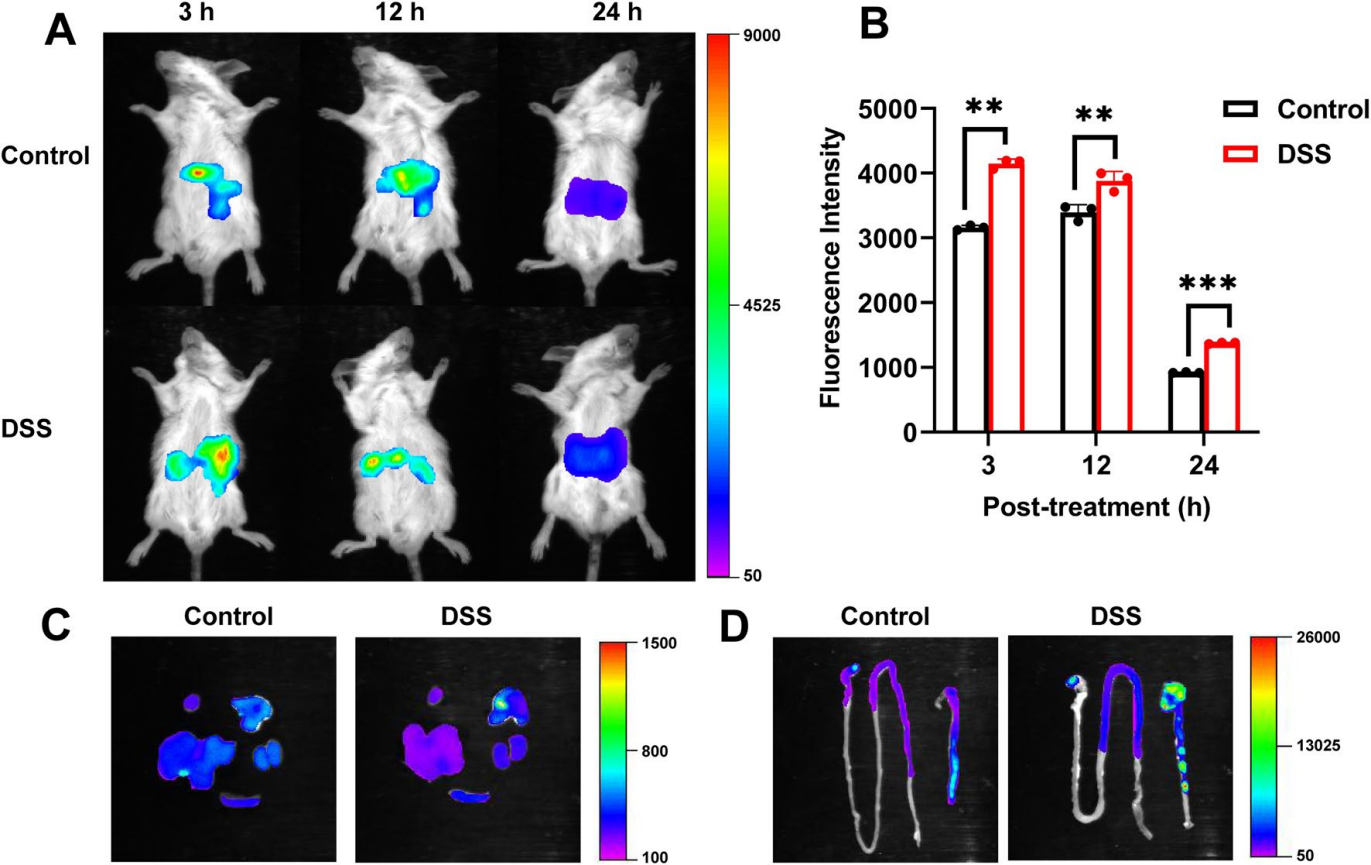
Biodistribution of DID NE@SA in mice with or without DSS-induced IBD. **A** DID fluorescence imaging of the stomach and intestines at different time points after oral administration of DID NE@SA, and **B** semi-quantitative analysis of the fluorescence intensity. Data are shown as mean ± SD (n = 3). *p < 0.05, **p < 0.01. In vitro imaging of DID fluorescence in **C** heart, liver, spleen, lung, kidney and **D** small intestine and colon 24 h after administration

It is worth mentioning that hydrogel has adhesion effect, which could increase the accumulation of mucosal penetration in the colon. In addition, negatively charged nanoparticles were more likely to preferentially target the IBD site due to the accumulation of positively charged proteins such as eosinophilic cationic proteins, transferrin, and antimicrobial peptides, which provided the advantage of the negatively charged CUR/EMO NE@SA targeting to colitis. To test this, near-infrared dye DID labeled NE@SA (DID NE@SA) was given orally to mice with or without DSS induced IBD to monitor the distribution for 24 h. Figure 4A showed the fluorescence images of mice after 3, 12, 24 h of administration. It was observed that DID NE@SA was present in the gut, and the fluorescence of IBD mice (DSS group) was significantly stronger than that of normal mice (Control group) at each time point. It summarized that the fluorescence intensity in the abdominal region to quantify the difference between the two groups (Fig. 4B). The average fluorescence intensity of the two groups decreased with time, and the fluorescence intensity of the DSS group was significantly higher than that of the Control group at each time point, which was consistent with the visible observation results. After 24 h oral administration, mice were sacrificed, and their hearts, liver, spleen, lungs and kidneys were collected for imaging, showing no obvious fluorescence distribution (Fig. 4C). The small intestines and colons of the DSS group showed stronger fluorescence than those of the Control group, indicating that the difference was statistically significant as confirmed by semi-quantitative analysis (Fig. 4D). These results confirmed that DID NE@SA could accumulate in inflamed colon tissue and had great potential in the treatment of IBD.

### Evaluation of treatment efficacy in mice with colitis

**Fig. 5:**
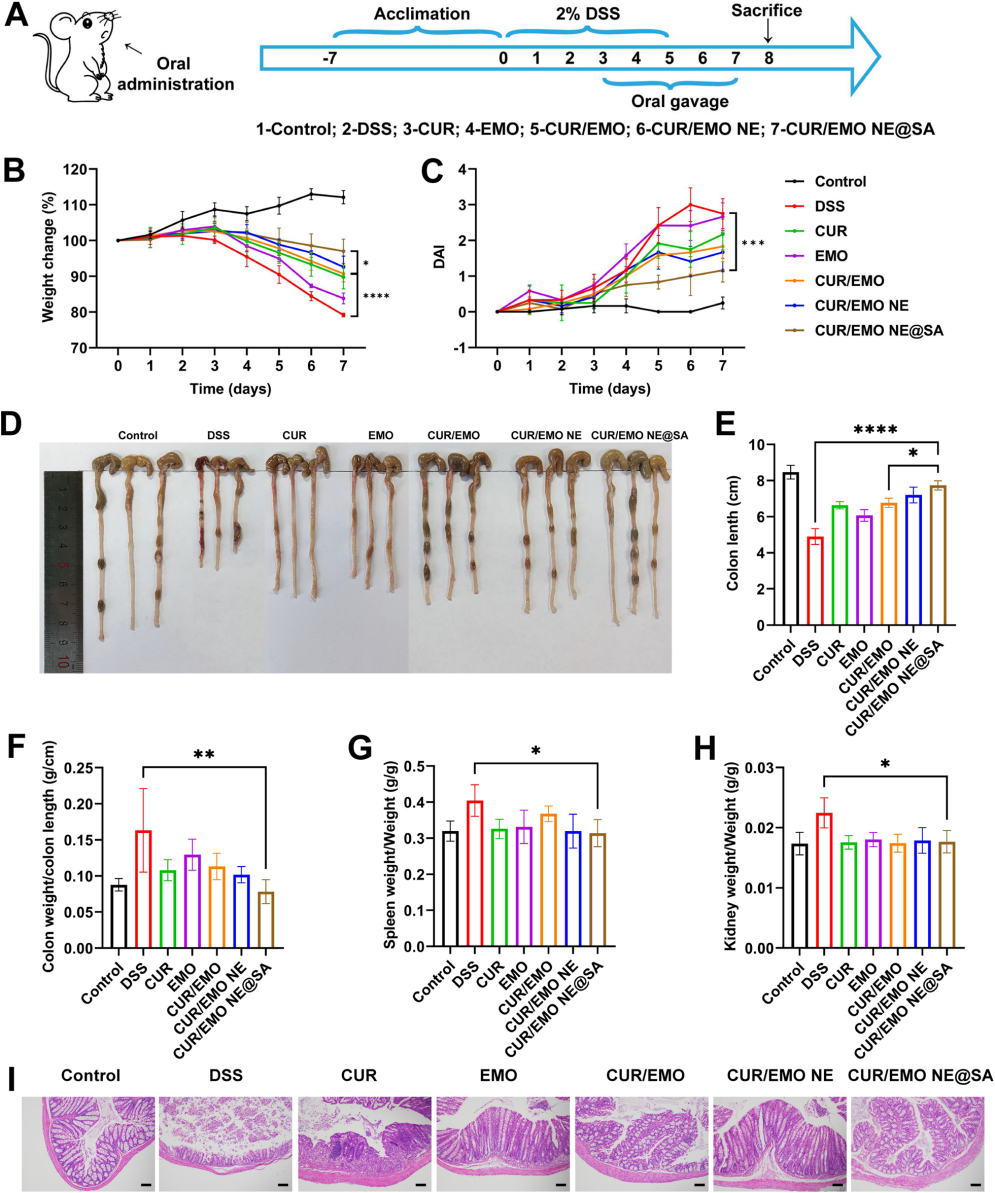
Evaluation of the in vivo treatment efficacy. **A** Schematic representation of the experimental procedure of IBD induction and oral gavage preparations in mice. **B** Weight change after different treatments and **C** disease index score. **D** Representative photographs of the colon from the different treatment groups. **E** Quantification of colon length, unit (cm), **F** colon weight divided by colon length, unit (g/cm), **G** weight divided by weight, unit (g/g), **H** kidney weight divided by weight, unit (g/g). Data are shown as mean ± SD (n = 3). *p < 0.05, **p < 0.01, ***p < 0.001, ****p < 0.0001. **I** HE staining of the colon in the different treatment groups. Scale bar were 100 μm

DSS-induced IBD mice were used to evaluate the in vivo therapeutic efficacy of CUR/EMO NE@SA (Fig. 5A). IBD was characterized by weight loss, diarrhea, and bloody stool, so we monitored weight and DAI, which was calculated based on weight change, fecal consistency, and the presence of blood in the stool to assess the severity of IBD. The higher the weight loss and DAI score, the more severe the IBD. The body weight of mice in the control group showed an increasing trend, and there was a significant difference between the CUR/EMO NE@SA group and the DSS group, indicating that the CUR/EMO NE@SA had a certain effect of weight recovery and pathological status improvement CUR/EMO NE@SA had better therapeutic efficacy than free drugs (Fig. 5B, C). A shortened colon was common result of damage to the epithelial barrier of the colon, mucosal damage, and dehydration of colitis tissue. According to colon photographs and length measurements (Fig. 5D, E), colon length and fecal integrity in the CUR/EMO NE@SA group had no significant difference with the control group, indicating that it significantly improved inflammatory in DSS-induced IBD mice. These results were indirectly confirmed by statistical analysis of unit colon weight (Fig. 5F). It is known that when inflammation occurs in an organism, large numbers of immune cells accumulate in the spleen and kidneys, increasing the weight of these organs. Compared to the water control group, spleen and kidney weights were significantly higher in the DSS control group, while there was no significant difference in spleen and kidney weights in the CUR/EMO NE@SA group (Fig. 5G, H). Hematoxylin and eosin staining of colon tissue are direct methods for the determination of inflammatory colon. Colonic tissue staining images of different mouse groups were shown in Fig. 5I. There was no inflammation or destruction of colon tissue in the healthy control group, while obvious inflammation was observed in the DSS model group, including necrosis of intestinal recess, reduction of cup cells, edema, increase of leukocyte infiltration, and irregular mucosal structure. In contrast, tissue inflammation levels were significantly reduced in the CUR/EMO NE@SA group, showing no obvious damaged mucosal structure. These results suggested that the CUR/EMO NE@SA could effectively ameliorate DSS induced IBD.

### In vivo anti-inflammatory and mucosal repair

**Fig. 6:**
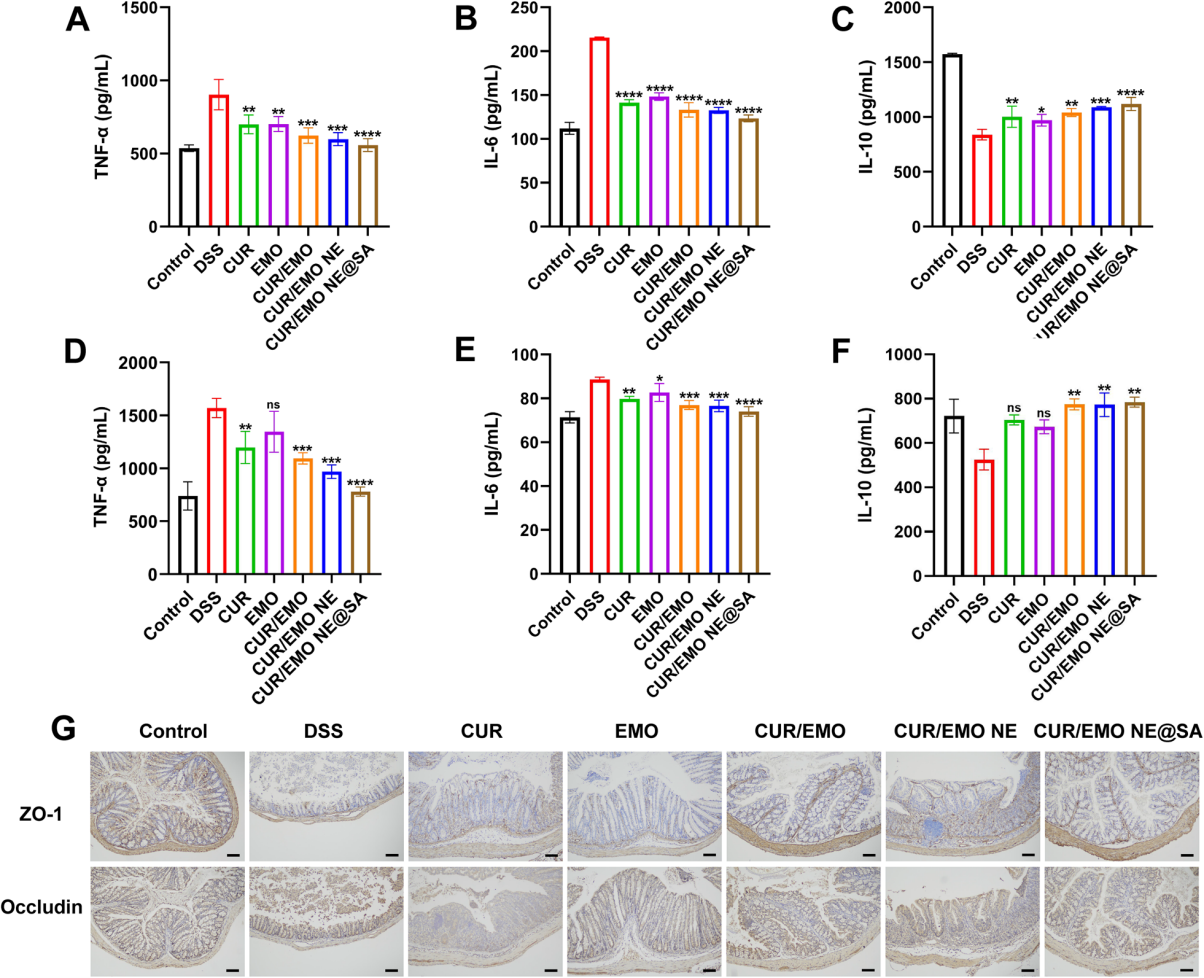
Anti-inflammatory and mucosal repair effects in vivo. **A**–**C** The amount of plasma TNF-α, IL-6, and IL-10 was measured by ELISA. **D**–**F** The amount of colon measured the TNF-α, IL-6 and IL-10 was measured by ELISA. *p < 0.05, **p < 0.01, ***p < 0.001, ****p < 0.0001 vs DSS group. Data are shown as mean ± SD (n = 3). **G** The expression of ZO-1 and occludin was detected by immunohistochemistry. Scale bar were 100 μm

The severity of inflammation was related to the number of inflammatory factors, so we measured the levels of inflammatory factors in plasma and colon of each group by ELISA [58]. As expected, the concentrations pro-inflammatory cytokines TNF-α and IL-6 of the plasma of the DSS group were 1567 pg/mL and 89 pg/mL, respectively, and the concentrations anti-inflammatory cytokines IL-10 were 625 pg/mL. While the TNF-α and IL-6 concentrations of plasma in the control group were 739 pg/mL and 71 pg/mL, respectively, and IL-10 was 722 pg/mL. The levels of IL-6 and TNF-α were significantly higher in the DSS model group than in the control group, and the levels of TNF-α and IL-6 in the CUR/EMO NE@SA group had no significant difference with those in the control group (Fig. 6A–C). Compared with the control group, the levels of TNF-α and IL-6 in part of the colon were significantly increased in the DSS model group, and the levels in the CUR/EMO NE@SA group had no significant difference (Fig. 6D–F). Therefore, the CUR/EMO NE@SA effectively reduced the concentration of pro-inflammatory cytokines in the serum and colon and increased the anti-inflammatory factor, thus exerting an anti-inflammatory effect.

As shown in Fig. 6G, compared with the normal group, the expression of ZO-1 and occludin in colon of DSS group decreased significantly, indicating that epithelial barrier function was significantly impaired after DSS treatment. However, the above two indicators expression increased in the colon after treatment with different formulations, and significantly increased in the CUR/EMO group compared with the free CUR and EMO groups. Moreover, the CUR/EMO NE@SA showed increased expression of ZO-1 and occludin, and the epithelial barrier structure was most complete. These results suggest that the CUR/EMO NE@SA had a best protective effect on epithelial cytoskeleton.

### Preliminary safety evaluation

**Fig. 7:**
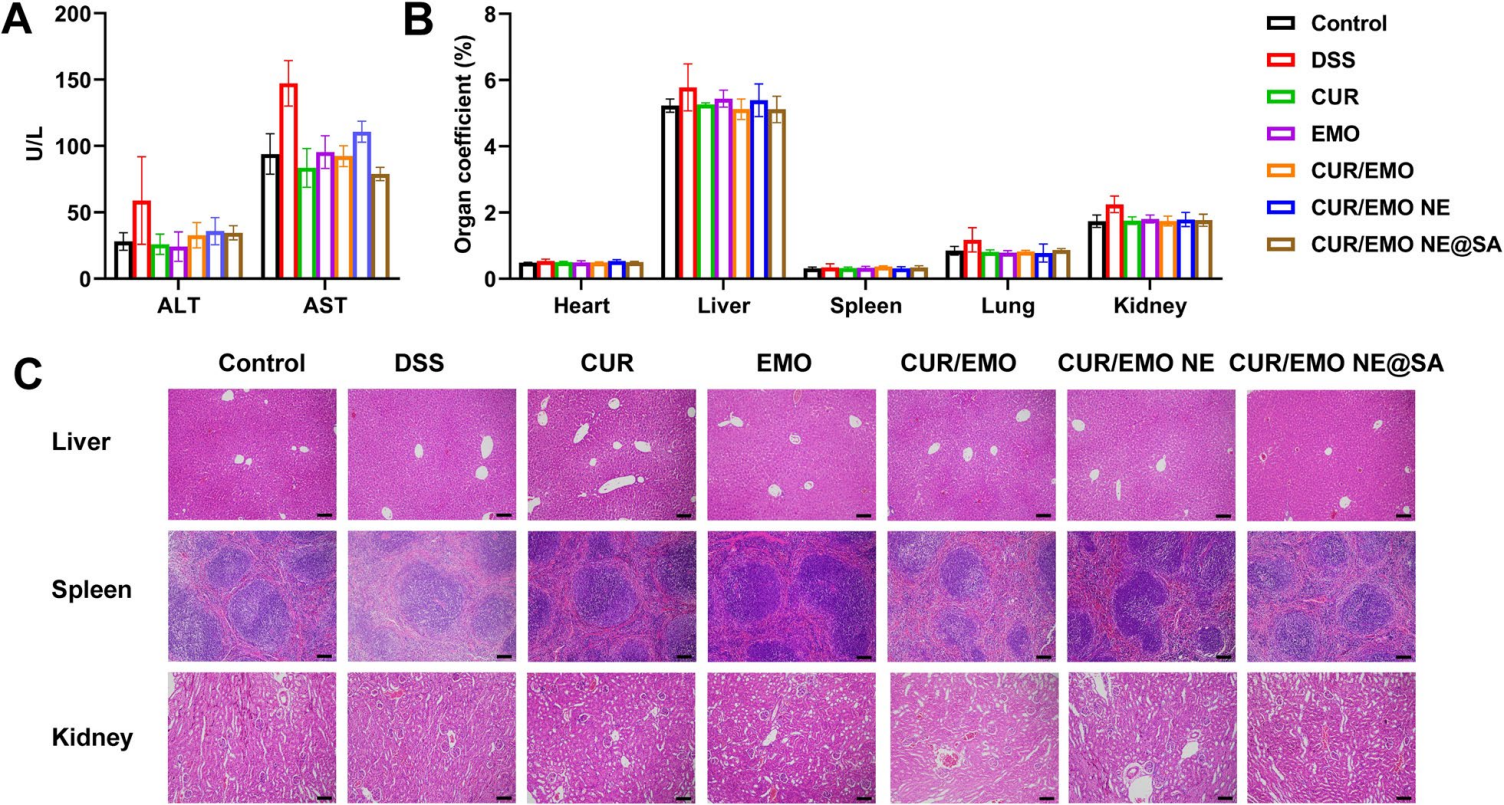
Drug safety evaluation. **A** The ALT and AST of the plasma. Data are expressed as the mean ± SD (n = 3). **B** Organ coefficient of the tissue. Organ coefficient = (organ weight/body weight) × 100%. Data are shown as mean ± SD (n = 3). **C** HE staining of liver, kidney and spleen. Scale bar were 100 μm

Histopathological examination by HE staining showed no pathological changes in liver, spleen and kidney after treatment (Fig. 7A). The ALT and AST of serum were detected to evaluate liver function, and it was found that the liver function of the preparation group had no obvious liver toxicity (Fig. 7B). The organ coefficients of heart, liver, spleen, lung and kidney were measured, and there was no significant difference in all formulations (Fig. 7C). These results illustrated the in vivo safety of the CUR/EMO NE@SA.

## Discussion

Much attention has also been paid to the field of controlled release of hydrogels, where drugs were uniformly dispersed in gel matrix and delivered in controlled, stable manner under certain conditions or over long periods of time. Hydrogel-coated nanoemulsion, mostly used to improve the stability of lipophilic drugs, percutaneous treatment of skin diseases, and sodium alginate hydrogel containing platelet-rich plasma for wound healing [59]. For example, chitosan hydrogel-coated NE form hydrogel-thickened nanoemulsions to overcome the low viscosity caused by NE for transdermal drug delivery [60]. Chitosan based oral gel was prepared by loading PVLO-SNEDDs into chitosan hydrogel to treat cold sores [61]. In addition, there are three-dimensional structures similar to hydrogels that electrospun fiber sponges for environmental treatment [62], and curved band structured multi-layered nanofiber membranes for air filtration [63]. To address air pollution, environmental friendly lignin shows the advantages of replacing fossil petroleum fuels [64], and hydrothermal technology can be applied to various plastics and biomass wastes [65]. Compared to DID, probes for Ni^2+^ is the combination of fluorescence imaging technology and fluorescent probes [66].

We constructed a pH-sensitive CUR/EMO NE@SA that released CUR and EMO slowly and specifically in the colon, helping to maintain therapeutic levels of the drug in circulation. During the progression of IBD, an excessive inflammatory response and damage to the mucosal lining of the gut was caused due to immune imbalance and excessive oxidative stress. A large number of activated macrophages, also known as M1 macrophages, were present at IBD site. These cells can continuously secrete a variety of pro-inflammatory cytokines to drive the progression of IBD. This makes macrophages to become potential target for inflammatory diseases. In our experiment, we explored the pharmacodynamics of CUR/EMO NE and the anti-inflammatory mechanisms of nanoparticles using LPS-activated macrophages as models of inflammation. CUR/EMO NE inhibited pro-inflammatory cytokines and promotes the expression of the anti-inflammatory cytokine more than other groups, indicating the cooperative anti-inflammation effect of CUR with EMO in the treatment of IBD.

After 24 h administration of DID NE@SA, the fluorescence intensity in DSS group was 1373.8, which was stronger than 917.5 of Control group, showing the significantly colonic accumulation of DID NE@SA in DSS group. The degree of colon accumulation of DSS group was much greater than that in the control group, which might due to the negative charge of the preparation could better adhered to the positively charged IBD site and improved its bioavailability. Therefore, the treatment efficiency of CUR and EMO co-delivery system could be improved and the side effects caused by systemic distribution could be reduced.

Our results further demonstrated that the CUR/EMO NE@SA had a certain therapeutic effect on DSS mice, and that the CUR/EMO combination had better therapeutic effect than the single drug, as observed by colon HE staining. The content of inflammatory cytokines in plasma and colon was measured by inflammatory cytokines. The nanoparticle could better reduce the pro-inflammatory cytokines TNF-α and IL-6, and increase the anti-inflammatory cytokines IL-10, indicating its excellent anti-inflammation effect. Immunohistochemical detection of ZO-1 and occludin also revealed that the co-delivery system showed better mucosal recovery, indicating that the combined encapsulating of CUR and EMO might have better therapeutic effect. These positive results were also due to the CUR/EMO NE@SA stability, high accumulation, and sustained drug release. As a result, side effects of the CUR/EMO NE@SA were reduced, which might enhance its’ clinical application.

## Conclusion

In this study, a pH-responsive CUR/EMO NE@SA was successfully developed, in which the CUR/EMO NE was loaded by chitosan and further crosslinked with sodium alginate. The CUR/EMO NE@SA could be thin films under increased shear or pressure to enable gavage with certain resistance to external forces and good rheological properties. It was stable in simulated gastric fluid, and collapsed in simulated colonic fluid, meaning it could successfully disintegrated in the colon. Importantly, the preparation could significantly alleviate and improve the colon inflammatory microenvironment by decreasing TNF-α and IL-6 expression, increasing IL-10 expression, scavenging reactive oxygen species in macrophages, and by ameliorating the restoration of intestinal mucosal tight junction protein expression. Furthermore, we revealed the molecular mechanism of the preparation for IBD treatment, which might due to the CUR and EMO synergic inhibition of NF-κB to improve the pro-inflammatory microenvironment. Our study provides a new IBD therapy strategy via synergically inhibiting inflammatory, repairing mucosal and clearing ROS by pH-sensitive hydrogel-encapsulated nanoemulsion drug delivery system, which might be developed for other chronic inflammatory disease treatment.

### Supplementary Information

**Additional file 1: Fig. S1.** In vitro anti-inflammatory and synergistic effects between CUR and EMO. **Fig. S2.** The expression of TNF-α was detected by qRT-PCR when CUR and EMO were 20 μg/mL in total. **Fig. S3.** The anti-inflammatory effects of CUR and EMO were investigated by detecting TNF-α expression by western blot. **Fig. S4.** Wound healing assay on colonic caco-2 cells to study the mucosal repair effects of CUR and EMO. **Fig. S5.** The mucosal restorative effect of the CUR and EMO in caco-2. **Fig. S6.** The protein levels of nuclear factor-κB (NF-κB) p65, phosphorylated NF-κB p65 (p-p65), IκBα, and phosphorylated IκBα (p-IκBα) were measured by western blot to study the inflammatory pathways in CUR and EMO. **Fig. S7.** Intuitive pictures of CUR/EMO NE on 1 days and 7 days. **Fig. S8.** The rheological characterization of CUR/EMO NE@SA. **Fig. S9.** Scanning electron micrographs of CUR/EMO NE@SA under different pH conditions. **Fig. S10.** In vitro cytotoxicity results by MTT assay. **Fig. S11.** Representative images of in vitro cell viability of RAW264.7 cells detected by Calcein-AM/PI staining. **Fig. S12.** DPPH antioxidant in vitro*.*

## Data Availability

All data generated or analyzed during this study are included in this article.

## Abbreviations

IBD: Inflammatory bowel disease
CUR: Curcumin
EMO: Emodin
IL-1β: Interleukin 1β
TNF-α: Tumor necrosis factor α
ZO-1: Zonula occludens 1
SA: Sodium alginate
CS: Chitosan
MCT: Medium chain fatty acids
DMEM: Dulbecco’s modified eagle’s medium
FBS: Fetal bovine serum
MTT: 3-(4, 5-Dimethylthiazol-2-yl)-2, 5-diphenyltetrazole bromide
LPS: Lipopolysaccharide
DSS: Dexosan sulfate sodium
ELISA: Enzyme-linked immunosorbent assay
SGF: Simulated gastric fluid
SIF: Simulated small intestine fluid
SCF: Simulated colonic fluid
Caco-2: Colonic carcinoma cells
PDI: Polydispersity index
TEM: Transmission electron microscope
SEM: Scanning electron microscopy
qRT-PCR: Quantitative real-time polymerase chain reaction
