# Supplementary material for: Oral hydrogel nanoemulsion co-delivery system treats inflammatory bowel disease via anti-inflammatory and promoting intestinal mucosa repair
Source: J Nanobiotechnology. 2023 Aug 18;21:275. doi: 10.1186/s12951-023-02045-4 (PMC10436423; doi:10.1186/s12951-023-02045-4)
Supplement: Supplementary file 1 — Additional file 1: Fig. S1. In vitro anti-inflammatory and synergistic effects between CUR and EMO. Fig. S2. The expression of TNF-α was detected by qRT-PCR when CUR and EMO were 20 μg/mL in total. Fig. S3. The anti-inflammatory effects of CUR and EMO were investigated by detecting TNF-α expression by western blot. Fig. S4. Wound healing assay on colonic caco-2 cells to study the mucosal repair effects of CUR and EMO. Fig. S5. The mucosal restorative effect of the CUR and EMO in caco-2. Fig. S6. The protein levels of nuclear factor-κB (NF-κB) p65, phosphorylated NF-κB p65 (p-p65), IκBα, and phosphorylated IκBα (p-IκBα) were measured by western blot to study the inflammatory pathways in CUR and EMO. Fig. S7. Intuitive pictures of CUR/EMO NE on 1 days and 7 days. Fig. S8. The rheological characterization of CUR/EMO NE@SA. Fig. S9. Scanning electron micrographs of CUR/EMO NE@SA under different pH conditions. Fig. S10. In vitro cytotoxicity results by MTT assay. Fig. S11. Representative images of in vitro cell viability of RAW264.7 cells detected by Calcein-AM/PI staining. Fig. S12. DPPH antioxidant in vitro. [file 12951_2023_2045_MOESM1_ESM.docx]

**Additional Information**

**Oral hydrogel nanoemulsion co-delivery system treats inflammatory bowel disease via anti-inflammatory and promoting intestinal mucosa repair**

Fenting Lei ^a#^, Fancai Zeng ^b#^, Xin Yu ^c#^, Yiping Deng ^a^, Zongquan Zhang ^d^, Maochang Xu ^d^, Nianhui Ding ^e^, Ji Tian ^a*^, Chunhong Li ^d*^

^a^ Analysis and Testing Center, School of Pharmacy, Southwest Medical University, Luzhou, Sichuan, 646000, China.

^b^ Laboratory of Biochemistry and Molecular Biology, School of Basic Medical Sciences, Southwest medical university, Luzhou 646000, China.

^c^ Chinese Pharmacy laboratory, School of Pharmacy, Southwest Medical University, Luzhou, Sichuan, China.

^d^ Department of Pharmaceutical Sciences, School of Pharmacy, Southwest Medical University, Luzhou, Sichuan, China.

^e^ School of Pharmacy, Southwest Medical University, Luzhou, 646000, China.


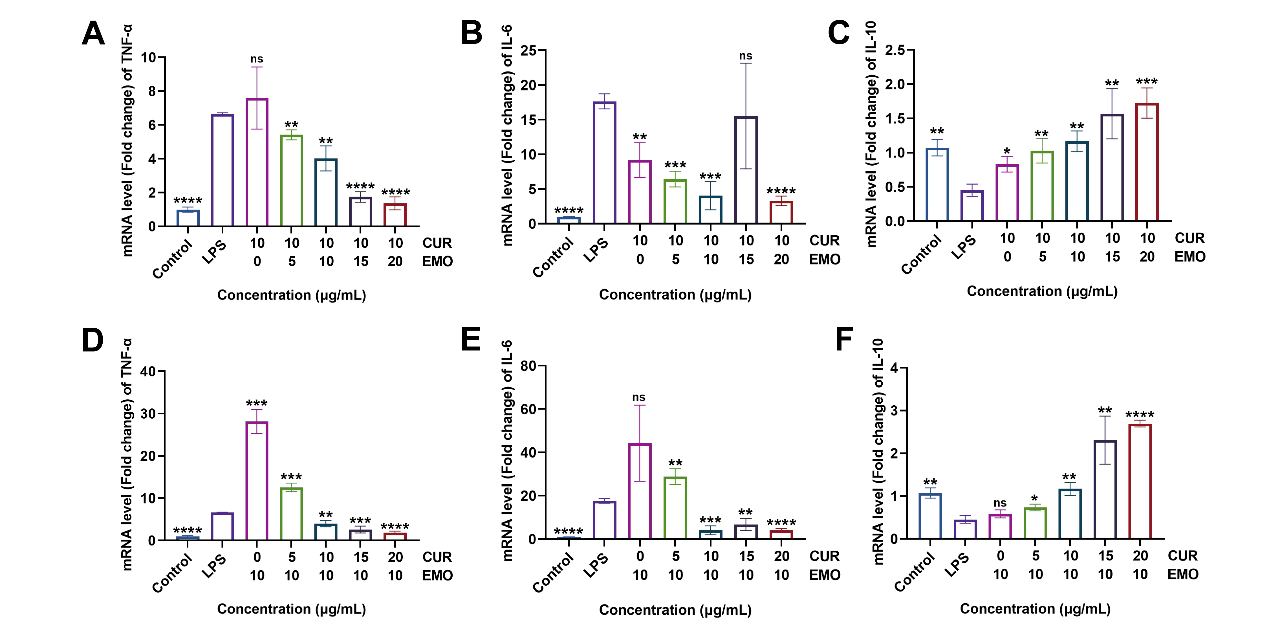


**Fig. S1.** *In vitro* anti-inflammatory and synergistic effects between CUR and EMO. (A-C) The expression of TNF-α, IL-6 and IL-10 was detected by qRT-PCR when the CUR was 10 μg/mL and the EMO was 0, 5, 10, 15, 20 μg/mL, respectively. (B-D) The expression of TNF-α, IL-6 and IL-10 was detected by qRT-PCR when the EMO was 10 μg/mL and the CUR was 0, 5, 10, 15, 20 μg/mL, respectively. *p < 0.05, **p < 0.01, ***p < 0.001, ****p < 0.0001 vs LPS group.


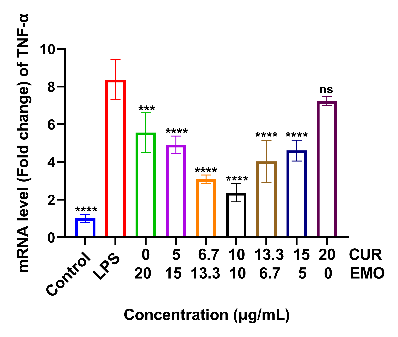


**Fig. S2.** The expression of TNF-α was detected by qRT-PCR when CUR and EMO were 20 μg/mL in total. *p < 0.05, **p < 0.01, ***p < 0.001, ****p < 0.0001 vs LPS group.


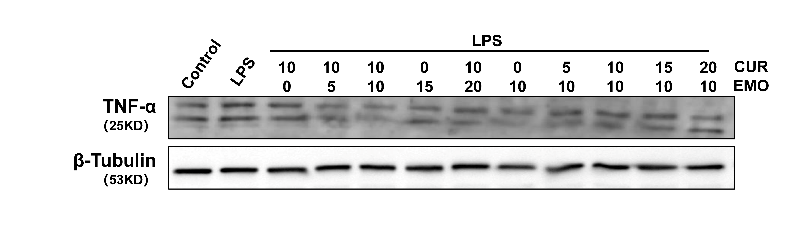


**Fig. S3.** The anti-inflammatory effects of CUR and EMO were investigated by detecting TNF-α expression by western blot.


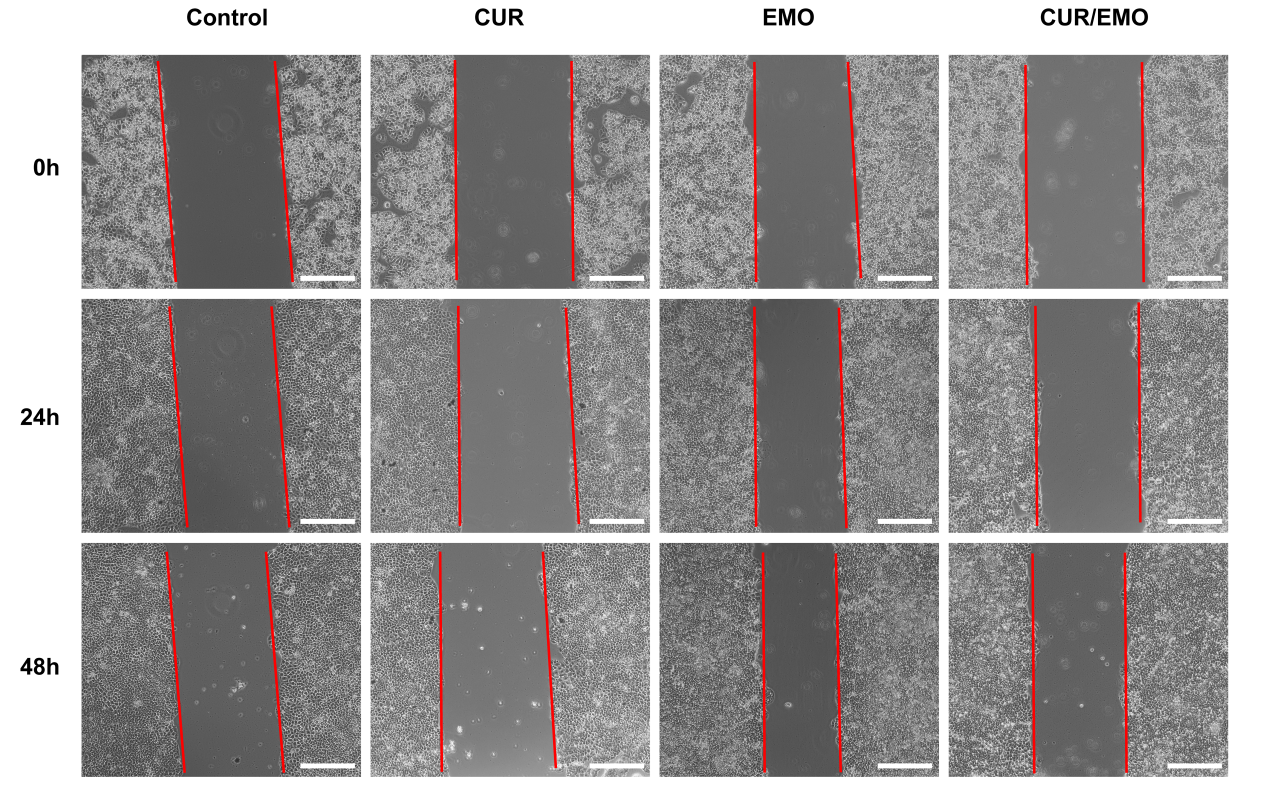


**Fig. S4.** Wound healing assay on colonic caco-2 cells to study the mucosal repair effects of CUR and EMO. Scale bar were 500 μm.


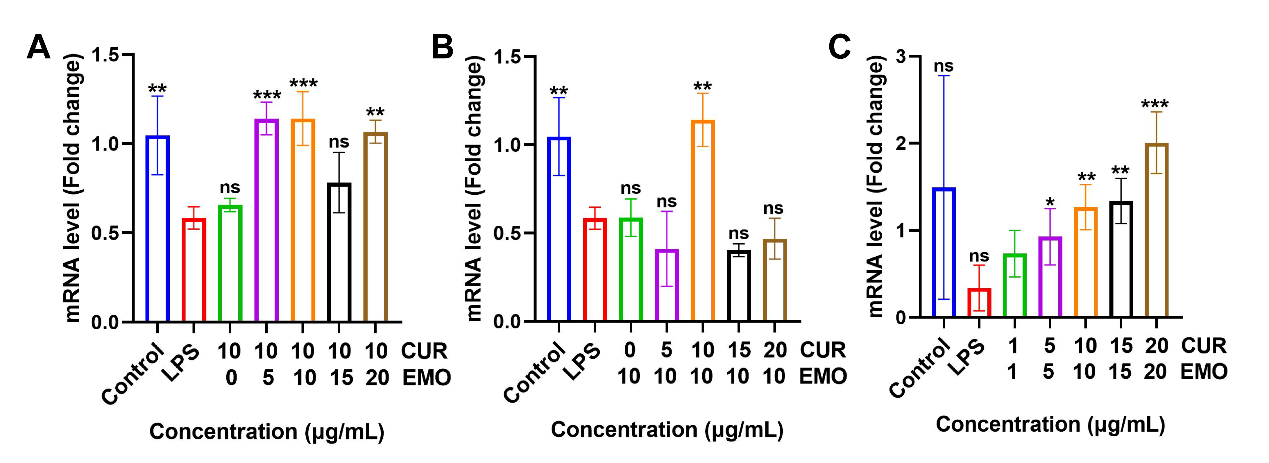


**Fig. S5.** The mucosal restorative effect of the CUR and EMO in caco-2. (A-C) The expression of the tight junction protein olaudin-1 was investigated by qRT-PCR. *p < 0.05, **p < 0.01, ***p < 0.001, ****p < 0.0001 vs LPS group.


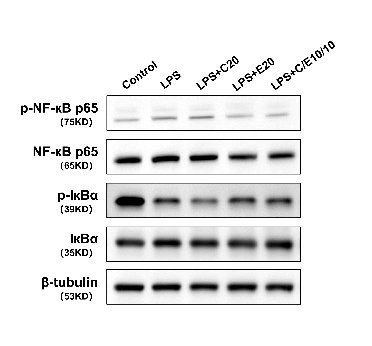


**Fig. S6.** The protein levels of nuclear factor-κB (NF-κB) p65, phosphorylated NF-κB p65 (p-p65), IκBα, and phosphorylated IκBα (p-IκBα) were measured by western blot to study the inflammatory pathways in CUR and EMO.


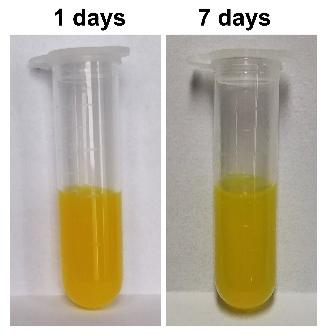


**Fig. S7.** Intuitive pictures of CUR/EMO NE on 1 days and 7 days.


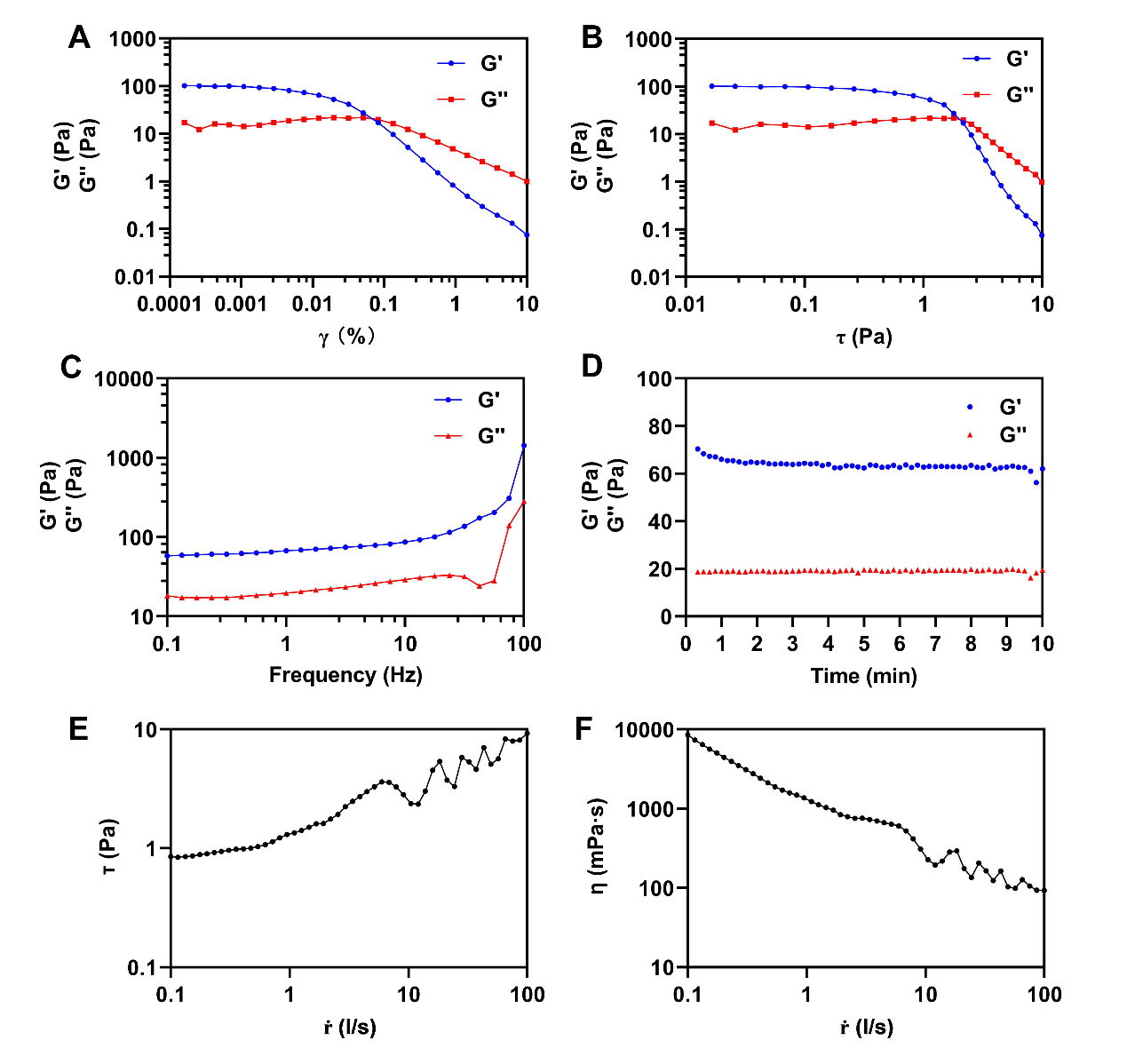


**Fig. S8.** The rheological characterization of CUR/EMO NE@SA. (A-B) The strain scanning, (C) angular frequency scanning, (D) time modulus profile, (E-F) the shear mode.


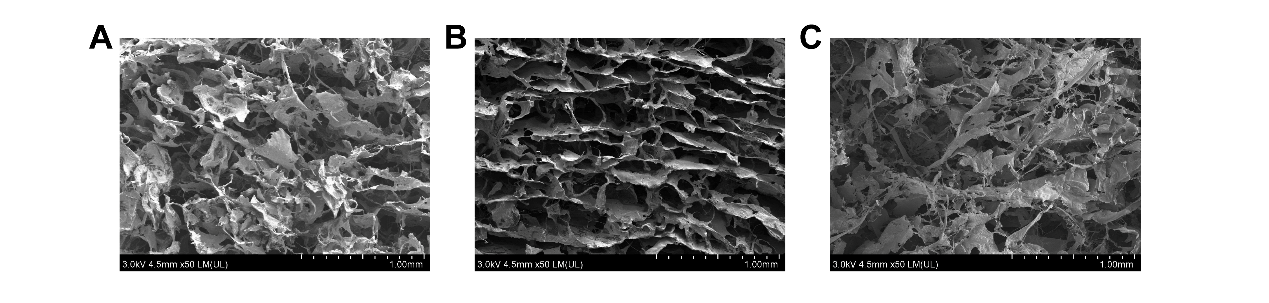


**Fig. S9.** Scanning electron micrographs of CUR/EMO NE@SA under different pH conditions. The hydrogel in (A) SGF (pH 1.2), (B) SIF (pH 6.8) (C) SCF (pH 7.8).


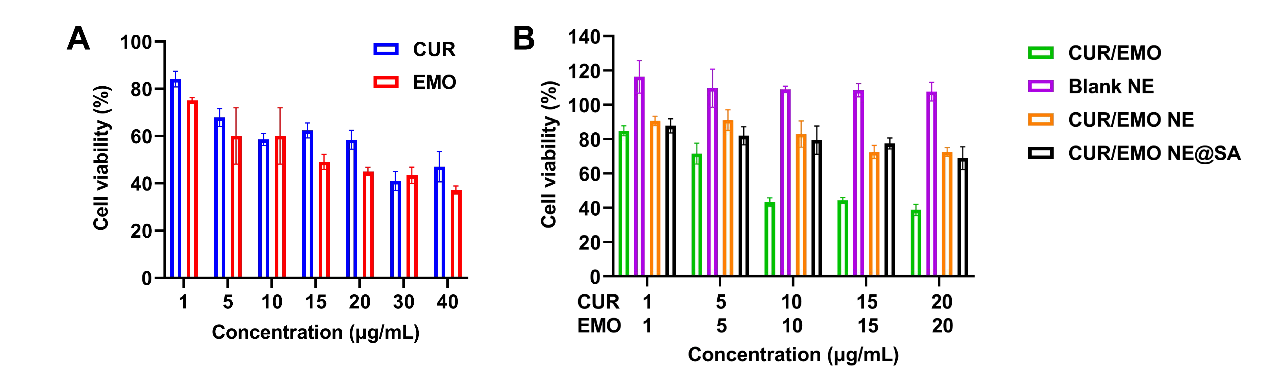


**Fig. S10.** *In vitro* cytotoxicity results by MTT assay. (A-B) RAW 264.7 cells were treated with CUR、EMO、CUR/EMO、Blank NE、CUR/EMO NE、CUR/EMO NE@SA for 24 h, and the cell viability was determined by MTT assay.


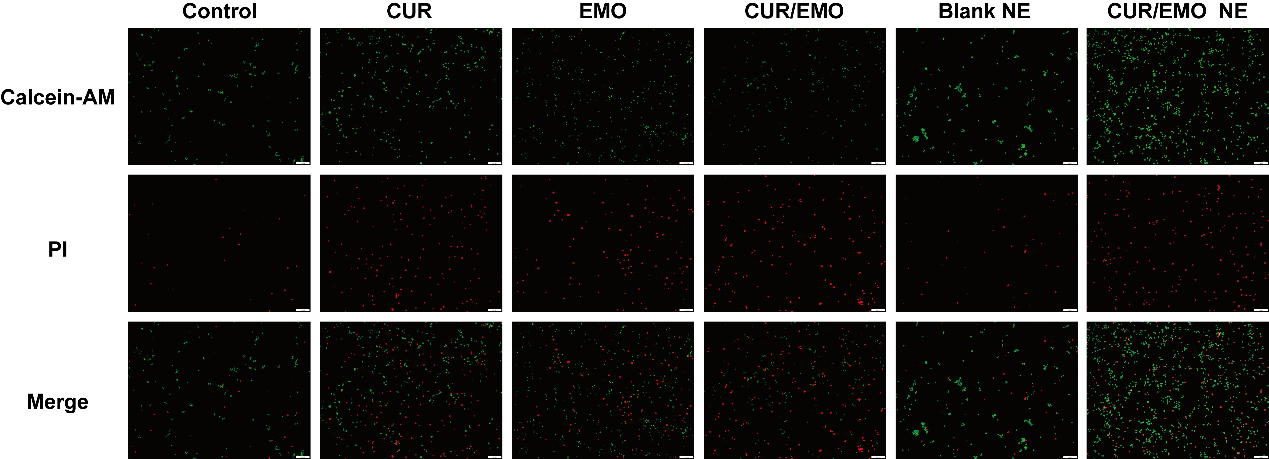


**Fig. S11.** Representative images of *in vitro* cell viability of RAW264.7 cells detected by Calcein-AM/PI staining. Green represents Calcein-AM fluorescence for live cells, red represents PI fluorescence for dead cells. Results are shown as mean ± SD (n = 3). Scale bar were 100 μm.


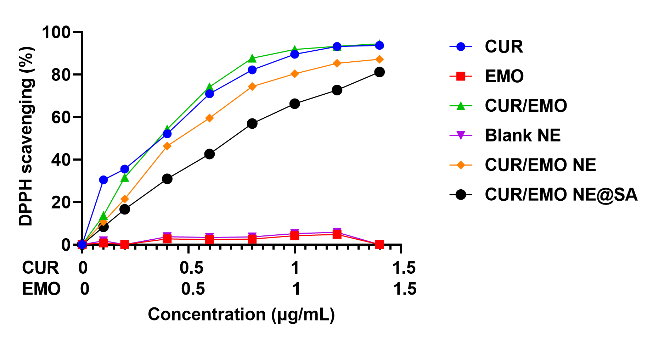


**Fig. S12.** DPPH antioxidant *in vitro.* The 200 μL of DPPH solution (0.04 mM) and CUR, EMO, CUR/EMO, Blank NE, CUR/EMO NE, CUR/EMO NE@SA were incubated for 30 min at room temperature in the dark, and the absorbance of the solutions was measured at 517 nm by multifunctional enzyme marker.
